# Regulation of the p38-MAPK pathway by hyperosmolarity and by WNK kinases

**DOI:** 10.1038/s41598-022-18630-w

**Published:** 2022-08-25

**Authors:** Zetao Liu, Wael Demian, Avinash Persaud, Chong Jiang, Arohan R. Subramanaya, Daniela Rotin

**Affiliations:** 1 Cell Biology Program, The Hospital for Sick Children, PGCRL 19-9715, 686 Bay St., Toronto, ON M5G 0A4 Canada; 2 Biochemistry Department, University of Toronto, Toronto, ON Canada; 3 Department of Medicine and Cell Biology, University of Pittsburgh, Pittsburgh, USA

**Keywords:** Biochemistry, Cell biology

## Abstract

p38-MAPK is a stress-response kinase activated by hyperosmolarity. Here we interrogated the pathways involved. We show that p38-MAPK signaling is activated by hyperosmotic stimulation in various solutions, cell types and colonic organoids. Hyperosmolarity sensing is detected at the level of the upstream activators of p38-MAPK: TRAF2/ASK1 (but not Rac1) and MKK3/6/4. While WNK kinases are known osmo-sensors, we found, unexpectedly, that short (2 h) inhibition of WNKs (with WNK463) led to elevated p38-MAPK activity under hyperosmolarity, which was mediated by WNK463-dependent stimulation of TAK1 or TRAF2/ASK1, the upstream activators of MKK3/6/4. However, this effect was temporary and was reversed by long-term (2 days) incubation with WNK463. Accordingly, 2 days (but not 2 h) inhibition of p38-MAPK or its upstream activators ASK1 or TAK1, or WNKs, diminished regulatory volume increase (RVI) following cell shrinkage under hyperosmolarity. We also show that RVI mediated by the ion transporter NKCC1 is dependent on p38-MAPK. Since WNKs are known activators of NKCC1, we propose a WNK**- > **NKCC1**- > **p38-MAPK pathway that controls RVI. This pathway is augmented by NHE1. Additionally, hyperosmolarity inhibited mTORC1 activation and cell proliferation. Thus, activation of p38-MAPK and WNKs is important for RVI and for cell proliferation.

## Introduction

In mammalian cells, the Mitogen Activated Protein Kinase (MAPK) proteins belong to three major classes of Ser/Thr kinases: ERK, JNK, and p38 MAPK (hereafter called p38). They orchestrate different cellular functions including proliferation/growth, differentiation, survival and stress response^1^. p38 is a stress response kinase that negatively regulates cell cycle progression at both the G1/S and G2/M transition phases by downregulation of cyclins and upregulation of CDK inhibitors^2,3^. Moreover, recent studies have shown that p38 plays an important role in coordinating cell size (mass) and cell cycle progression in animal cells: small cells display elevated p38 activity and spend more time in G1 phase than larger cells, an effect lost upon inhibition of p38^4^.

Incubating cells in hyperosmotic solution leads to cell shrinkage followed by volume recovery by a process called regulatory volume increase (RVI)^5,6^. In the absence of bicarbonate, RVI is propelled by influx of Na^+^ and Cl^−^, mediated by ion transporters such as the Na^+^/H^+^ antiporter (NHE1 or 2) or the cotransporter Na^+^/K^+^/2Cl^−^ (NKCC1 or 2), followed by water^5–8^. Interestingly, hyperosmotic stress also activates p38^9–11^, and p38 was shown to be essential for RVI^12,13^

Our recent work demonstrated that NKCC1 provides a link between cells mass and cell volume regulation by regulating both cell volume and mTORC1 activation^14^. Moreover, we showed that stable knockdown of NKCC1 led to reduced cell size/volume, inhibition of p38^14^ and impaired RVI. We thus hypothesized that p38 may mediate the positive effect of NKCC1 on RVI.

In addition to p38, WNK kinases (the upstream activators of SPAK/OSR1) are also known to be stimulated by hyperosmolarity-induced cell shrinkage and to regulate RVI^15–17^. However, the relationship between p38 and WNK -mediated osmo-sensing and contribution to RVI is/are unknown.

Thus, given the importance of p38 in regulating cell volume, cell mass and cell cycle, we investigated the mechanism and pathways responsible for the regulation of p38 by hyperosmolarity, and the possible link between p38 and the WNK kinases, especially in regulating RVI. Our work identified the upstream hyperosmotic sensors of p38 activation, and provides a surprising finding that a short-term exposure to hyperosmolarity leads to WNK kinases-mediated *suppression* of p38, via regulation of TAK1 or TRAF2/ASK1. This suppression is transient, however, as long-term inhibition of WNK inhibits p38, supporting the finding that both p38 and WNK work in concert (or sequentially, possibly via a WNK- > NKCC1- > p38 pathway) to promote RVI. Moreover, hyperosmolarity severely reduces cell proliferation likely by suppressing mTORC1. WNK inhibition also attenuates cell proliferation, while p38 inhibition has only a small effect, likely due to its maintenance of high mTORC1 activity.

## Results

### Ion requirement for the activation of p38-MAPK by hyperosmolarity

**Figure 1:**
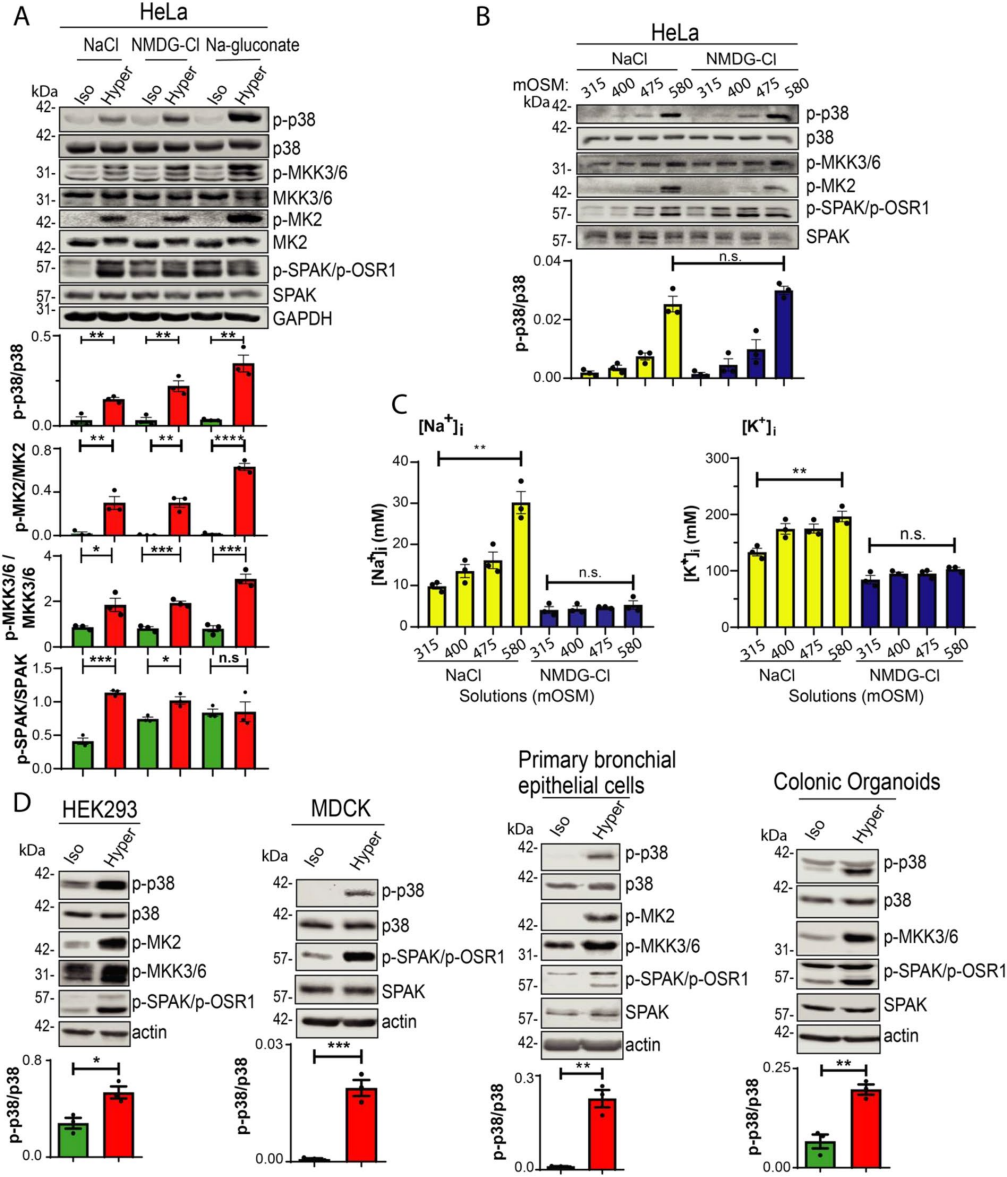
p38 is activated following hyperosmotic treatment. (**A**) HeLa cells were treated with 315 mOSM Iso-osmotic (Iso) or 580 mOSM Hyper-osmotic (Hyper) solutions of NaCl, NMDG-Cl^−^ or Na^+^-gluconate for 15 min. (**B**) HeLa cells were treated with either NaCl or NMDG-Cl^−^ solutions with increasing osmotic concentrations, as indicated. (**C**) Quantitation of intracellular Na^+^ or K^+^ concentration ([Na^+^]_i_ and [K^+^]_i_) in HeLa cells following treatment with either NaCl or NMDG-Cl^−^ solutions with escalating osmotic concentrations. (**D**) HEK293, MDCK, Human Primary Bronchial Epithelial cells or mouse Colonic Organoids were treated with iso-osmotic or hyper-osmotic NaCl for 15 min. Quantification of active (phosphorylated) p38 (p-p38), MK2 (p-MK2) or MKK3,6 (p-MKK3/6) relative to their respective total proteins is depicted below their respective blots. All data are mean ± s.e.m, *N* = 3 independent experiments, *p*-values: Not significant (n.s.) > 0.05; * < 0.05; ** < 0.01; *** < 0.001; **** < 0.0001. *p*-value were calculated using Student’s *t*-test.

It was previously shown that hyperosmolarity activates p38^9–11^, as we confirm here as well (Fig. 1A). To interrogate the ionic requirement for the activation of p38 by hyperosmotic stress, we incubated HeLa cells with hyperosmotic (or iso-osmotic control) solutions composed of NaCl, NMDG-Cl^−^, Na^+^-Gluconate, or Choline-Cl^−^, each at 270 mM (580 mOsm), compared to 135 mM (315 mOsm) in the iso-osmotic controls. Our results show that all these hyperosmotic solutions were able to activate p38 (p-p38) and its downstream target MK2 (p-MK2) (Fig. 1A and Figure S1A) with escalating osmotic stress increasing p38 activation to the same extent in both NaCl and NMDG-Cl solutions (Fig. 1B). This activation is similar between the NaCl and NMDG-Cl solutions, despite the fact that intracellular Na^+^ concentrations ([Na^+^]_i_) is ~ 30 mM in the 580 mOsm NaCl solution, versus. ~ 5 mM [Na^+^]_i_ in the 580 mOsm NMDG-Cl solution (Fig. 1B,C). A similar activation of p38 by hyperosmotic NaCl solution was observed in HEK293 and MDCK cells, as well as in primary bronchial epithelial cells and in colonic organoids (Fig. 1D). These results suggest that Na^+^ itself is not necessary for the activation of p38 by hyperosmolarity.

### Signaling pathway responsible for hyperosmotic activation of p38

The canonical pathway for activation of p38 includes dual phosphorylation on tyrosine and threonine residues on a conserved TGY motif of p38, via phosphorylation by MKK3, MKK6, or MKK4 kinases^3,18–21^. Hence, we tested the effect of hyperosmolarity on the p38 upstream activators MKK3/MKK6 and MKK4 in HeLa cells. Our data show that active, phosphorylated p-MKK3/p-MKK6 and p-MKK4 levels were all elevated in hyperosmotic solutions (NaCl, NMDG-Cl^-^, or Na^+^-Gluconate) in HeLa cells, as also observed (for p-MKK3/p-MKK6) in HEK293 cells, Human Primary Bronchial Epithelial Cells and in colonic organoids (Fig. 1A,D, and Figure S1B).

**Figure 2:**
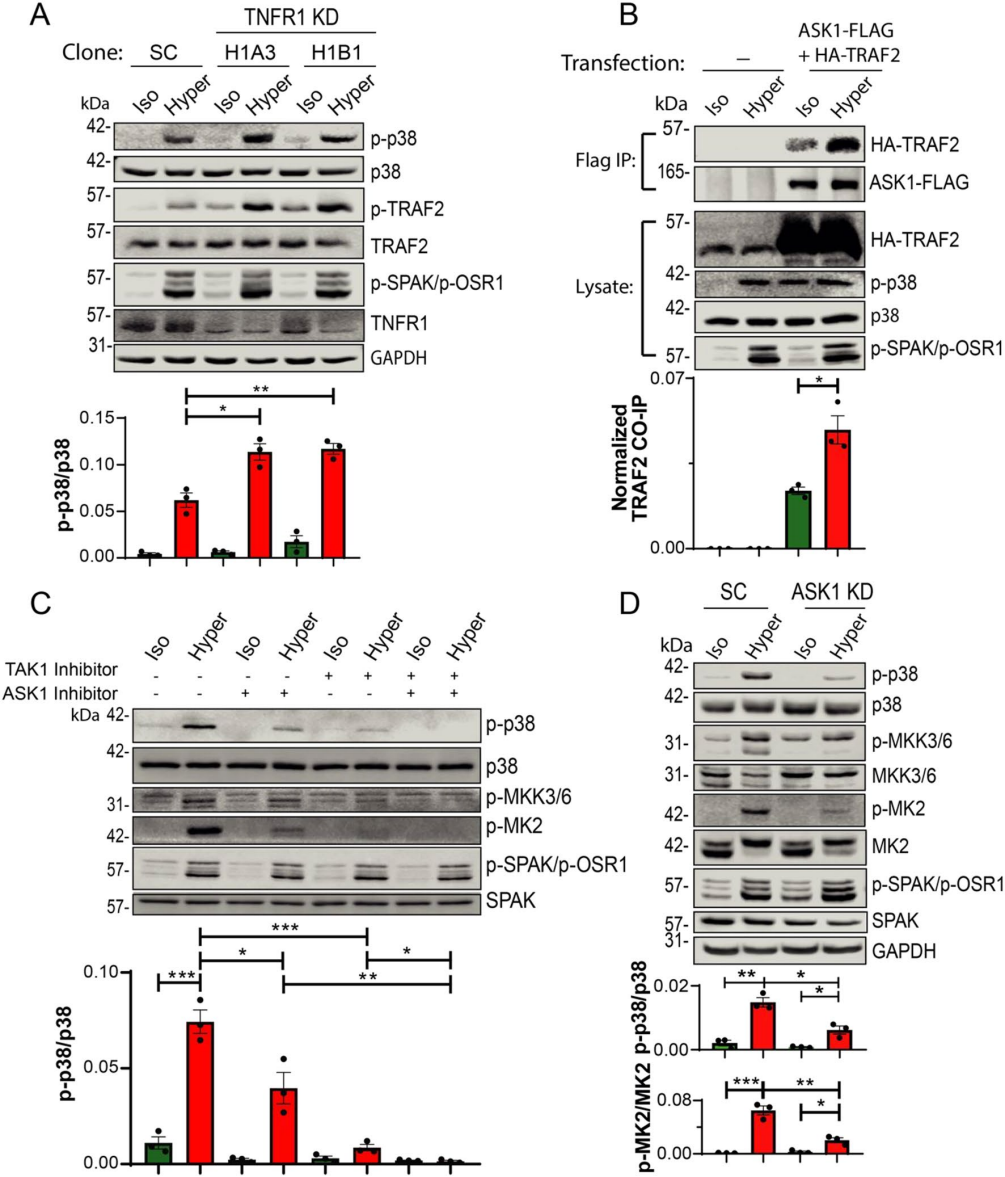
MAP3Ks, ASK1 and TAK1 induce p38 activation in HeLa cells upon hyperosmotic treatment. (**A**) Scramble Control (SC) or two different TNFR1 stable knockdown HeLa cell lines (H1A3, H1B1) were treated with iso-osmotic (Iso) or hyper-osmotic (Hyper) NaCl solutions for 15 min. (**B**) HeLa cells were transfected (or not) with ASK1-FLAG and HA-TRAF2 for 48 h and treated with iso- or hyper- osmotic NaCl solutions. ASK1-FLAG was immunoprecipitated with FLAG affinity beads and TRAF2 co-immunoprecipitation was determined by immunoblotting for TRAF2. (**C**) HeLa cells were treated (or not) with 10 µM GS-444217 (ASK1 inhibitor) for 2 h, 1 µM 5Z-7-Oxozeanol (TAK1 inhibitor) for 1 h, or both inhibitors, followed by incubation with iso- or hyper- osmotic NaCl solutions for 15 min in the absence/presence of GS-444217 or 5Z-7-Oxozeanol, where indicated. Quantification of active p38 (p-p38) as well as co-immunoprecipitated (Co-IP) TRAF2 is depicted below their respective blots. (**D**) Inhibition of p38 signaling in ASK1 knockdown (KD) HeLa cells relative to scramble control (SC) cells under hyper-osmotic stress. Experiment was done similar to panel A. Quantification of active p38 (p-p38), MK2 (p-MK2) or co-immunoprecipitated (Co-IP) TRAF2 is depicted below their respective blots. All data are mean ± s.e.m, *N* = 3 independent experiments. Statistics as described in Fig. 1.

To investigate hyperosmotic sensitivity of upstream activators of MKK3, MKK6 and MKK4 kinases, we analyzed activation of TRAF2 and its binding partner ASK1 (MAP3K5) in response to hyperosmotic stress. Our results show that TRAF2 is phosphorylated and activated in hyperosmotic solutions (Fig. 2A). Moreover, the association of (transfected) ASK1 with TRAF2 was enhanced under such elevated osmolarity (Fig. 2B). (We were not able to detect association of endogenous ASK1 and TRAF2, likely due to the inability of available antibodies to detect their low endogenous expression in our cells). Inhibition of ASK1 (with GS-444217), which is essential for TRAF2/MAPK activation^22,23^ suppressed the p38 activation observed under hyperosmotic condition (Fig. 2C), as was also observed in ASK1 (stably) knocked-down cells (Fig. 2D), despite exhibiting only a partial level of knockdown (Figure S1C). In contrast to a previous study^24^, inhibition of Rac1, an upstream activator of MKK3, did not block activation of p38 by elevated osmolarity in our experiments (Figure S1D).

TRAF2 and ASK1 are known activators of p38 downstream of active TNFα receptor^3,21,25^. Interestingly, knockdown of TNFRα (TNFR1) not only did not suppress p38 activation under hyperosmotic conditions, but actually enhanced it (Fig. 2A), suggesting that the TNFRα is not responsible for activation of p38 in hyperosmotic environment.

**Figure 3:**
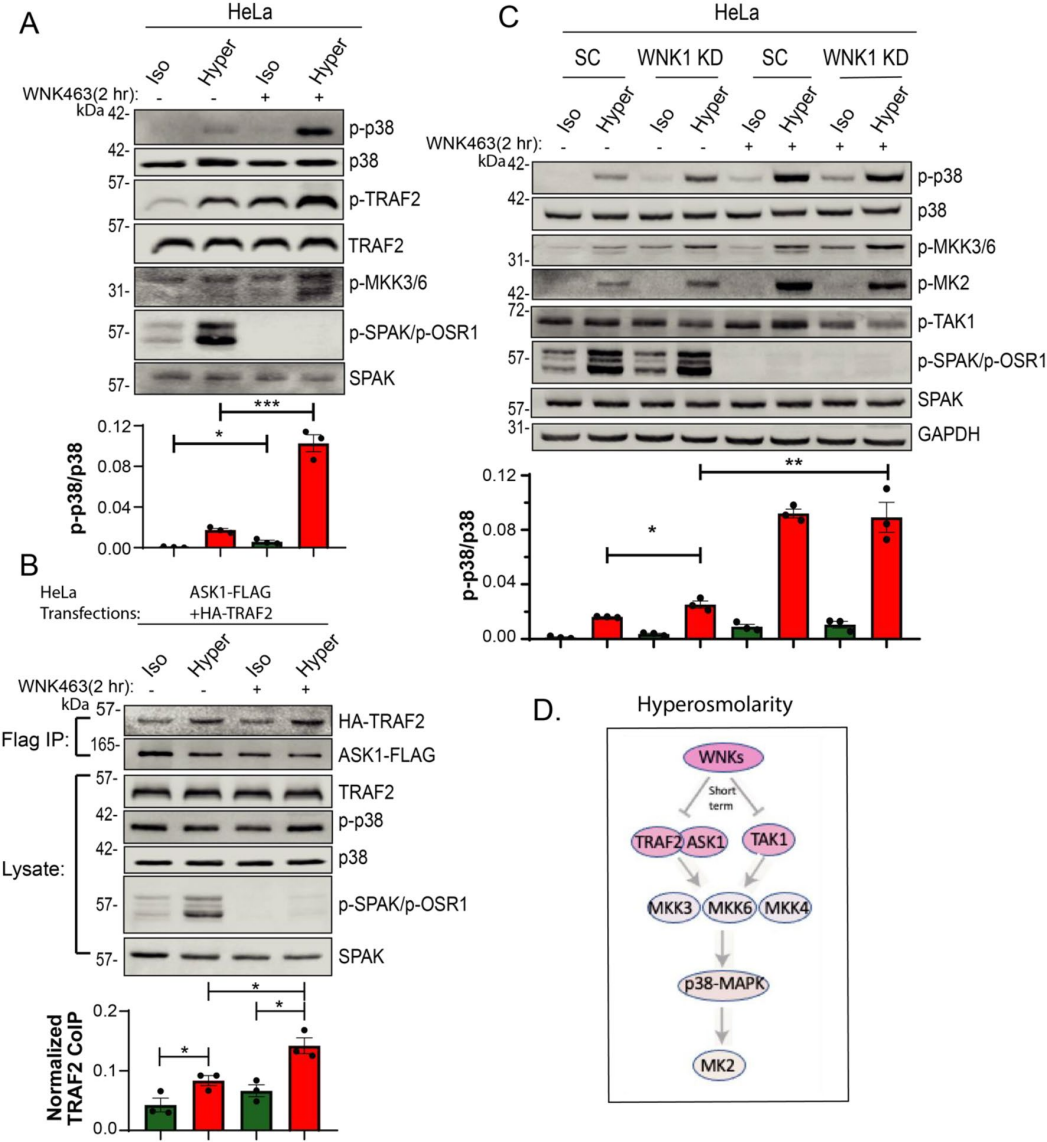
Short-term inhibition of WNK kinases, or WNK1 depletion, induces ASK1/TRAF2 interactions and p38 activation. (**A**) HeLa cells were pretreated without or with 10 μM pan-WNK inhibitor (WNK463) for 2 h followed by incubation with iso- or hyper- osmotic NaCl solutions for 15 min in the absence/presence of WNK463. (**B**) HeLa cells were transfected with ASK1-Flag and HA-TRAF2 for 48 h and then treated with 10 μM WNK463 for 2 h, followed by incubation in either iso- or hyper- osmotic NaCl solutions for 15 min in the absence/presence of WNK463. ASK1-FLAG was immunoprecipitated with FLAG affinity beads and TRAF2 co-immunoprecipitation was determined by immunoblotting for TRAF2. (**C**) WNK1 stable knockdown (or scramble control, SC) in HeLa cells were treated (or not) with 10 μM WNK463 for 2 h followed by incubation with the indicated NaCl solutions for 15 min in the absence/presence of WNK463. Quantification of active p38 (p-p38) or co-immunoprecipitated TRAF2 is depicted below their respective blots. All data are mean ± s.e.m, *N* = 3 independent experiments. Statistics as described in Fig. 1. (**D**) A model depicting a short-term WNKs-mediated suppression of the p38 pathway under hyperosmotic conditions.

Collectively, our experiments revealed that the TRAF2/ASK1-MKK3/6/4-p38-MK2 pathway is a hyperosmotic—sensitive signaling pathway in several types of mammalian cells (Fig. 3D).

### Short term WNK inhibition stimulates p38 activity

It is well-established that the WNK kinases and their downstream kinase substrates, SPAK and its relative OSR1, are activated by hyperosmotic stress^26^, as also shown in Fig. 1.

To test whether WNK kinases affect p38 activation by hyperosmolarity, we treated cells with WNK463, a pan-WNK-kinase inhibitor that inhibits WNK1-4^27^ for 2 h. Our results show that such WNK463 treatment significantly *enhanced* p38 activation under hyperosmotic conditions in several cell types, including HeLa cells (Fig. 3A), primary bronchial epithelial cells, and colonic organoids (Figure S1E,F). WNK463 treatment (2 h) also enhanced TRAF2/ASK1 interaction (Fig. 3B), a step important for p38 activation. Together, these results suggest that WNK normally inhibits p38 activation.

**Figure 4:**
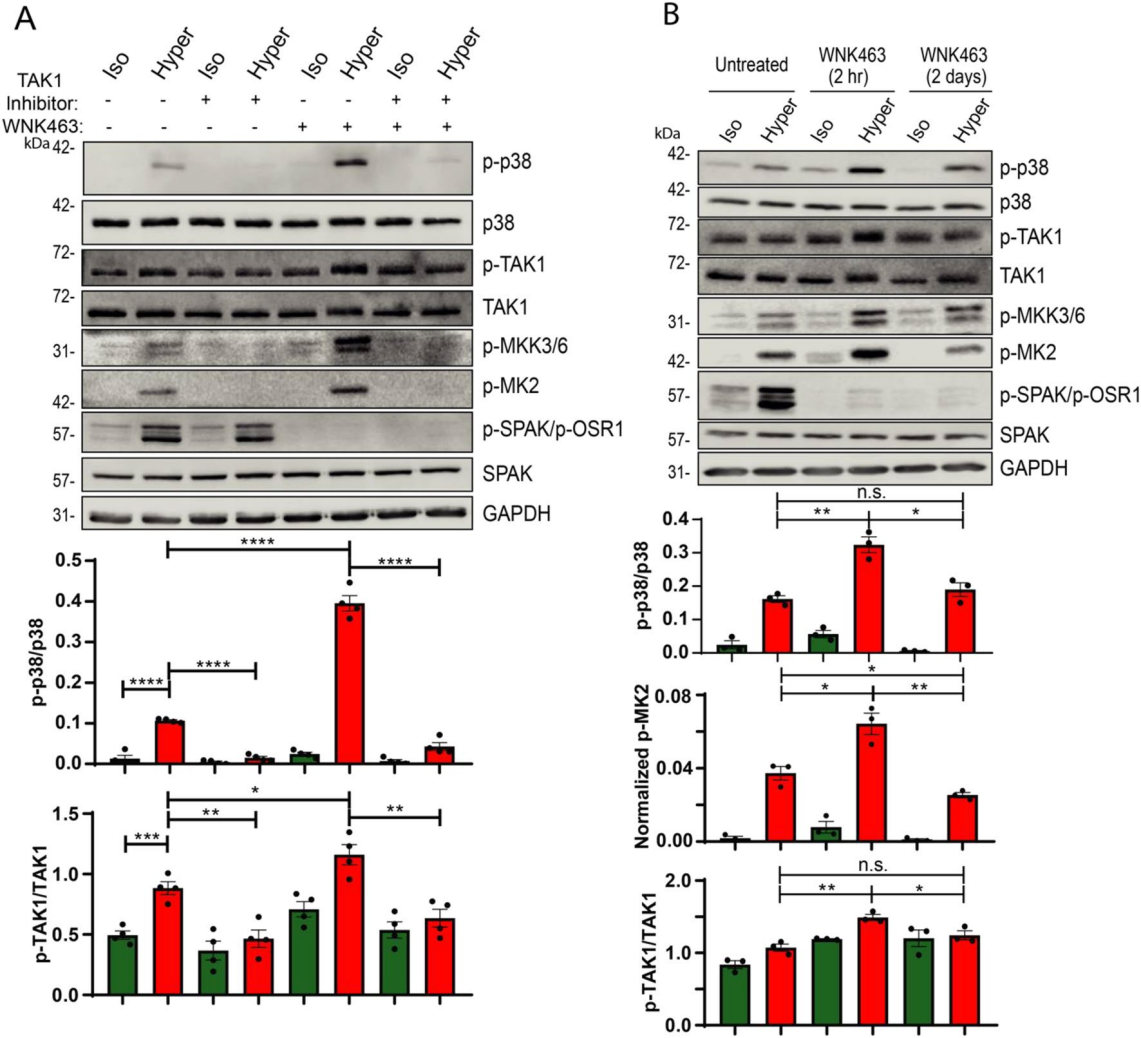
WNK kinases inhibit the p38 pathway by suppressing TAK1. (**A**) HeLa cells were treated (or not) with 1 μM 5Z-7-Oxozeanol (TAK1 inhibitor) for 1 h or 10 μM WNK463 (pan-WNK inhibitor) for 2 h, or both inhibitors, followed by incubation with iso- or hyper- osmotic NaCl solutions for 15 min. (**B**) HeLa cells were treated (or not) with 10 μM WNK463 for 2 h or 2 days followed by incubation with either iso- or hyper-osmotic NaCl solutions for 15 min. Quantification of active p38 (p-p38), TAK1 (p-TAK1) or MK2 (p-MK2) is depicted below their respective blots. All data are mean ± s.e.m, *N* ≥ 3 independent experiments. Statistics as described in Fig. 1.

In accord, strong enhancement of p38 activation was found in HeLa cells in which WNK1 was stably knocked-down (KD) with shRNA (Fig. 3C), suggesting that WNK1 is a major contributor to p38 regulation. In agreement with the ability of WNK463 to enhance p38 activation, it also enhanced the activity of the upstream (MKK3/MKK6) and downstream (MK2) effectors of p38 (Figs. 3C,4A). Collectively, these results suggest that WNK kinases normally suppress the p38 signaling pathway under hyperosmotic conditions (Fig. 3D).

To elucidate the possible mechanism(s) by which WNKs inhibit p38 activation, we focused on TAK1, a kinase that under immune stimulation activates the p38 pathway^28^, and is inhibited by WNK1^29^. As seen in Fig. 4A, the strong stimulation of the p38 pathway (MKK3/6-p38-MK2) by WNK463 under hyperosmotic conditions was almost completely abolished upon inhibition of TAK1 with 5Z-7-Oxozeaenol, a known inhibitor of TAK1^30^. As indicated above, WNK463 also increased the association of TRAF2 with ASK1 (Fig. 3B).

These results suggest that WNKs inhibit the p38 pathway by suppressing TAK1, an activator of MKK3/6 and hence of the p38 pathway, as well as by reducing TRAF2/ASK1 interactions (Fig. 3D).

While a short term (2 h) WNK463 treatment stimulated p38 activity, we found that a long term WNK463 treatment actually *attenuated* activity of p38 (Fig. 4B) a relevant step in the regulation of RVI, described below.

### Regulation of RVI by p38 vs WNK kinases

Both p38 and WNK kinases are known osmo-sensors activated by hyperosmolarity, which promote RVI. This raises the question as to how they can both promote RVI if WNKs inhibits p38. To address this potential contradiction, we analyzed the contribution of p38, WNKs, or both, to RVI in our HeLa cells, the time course of their effect on RVI, and the correlation of the RVI time course to the time course of WNK-mediated p38 inhibition.

**Figure 5:**
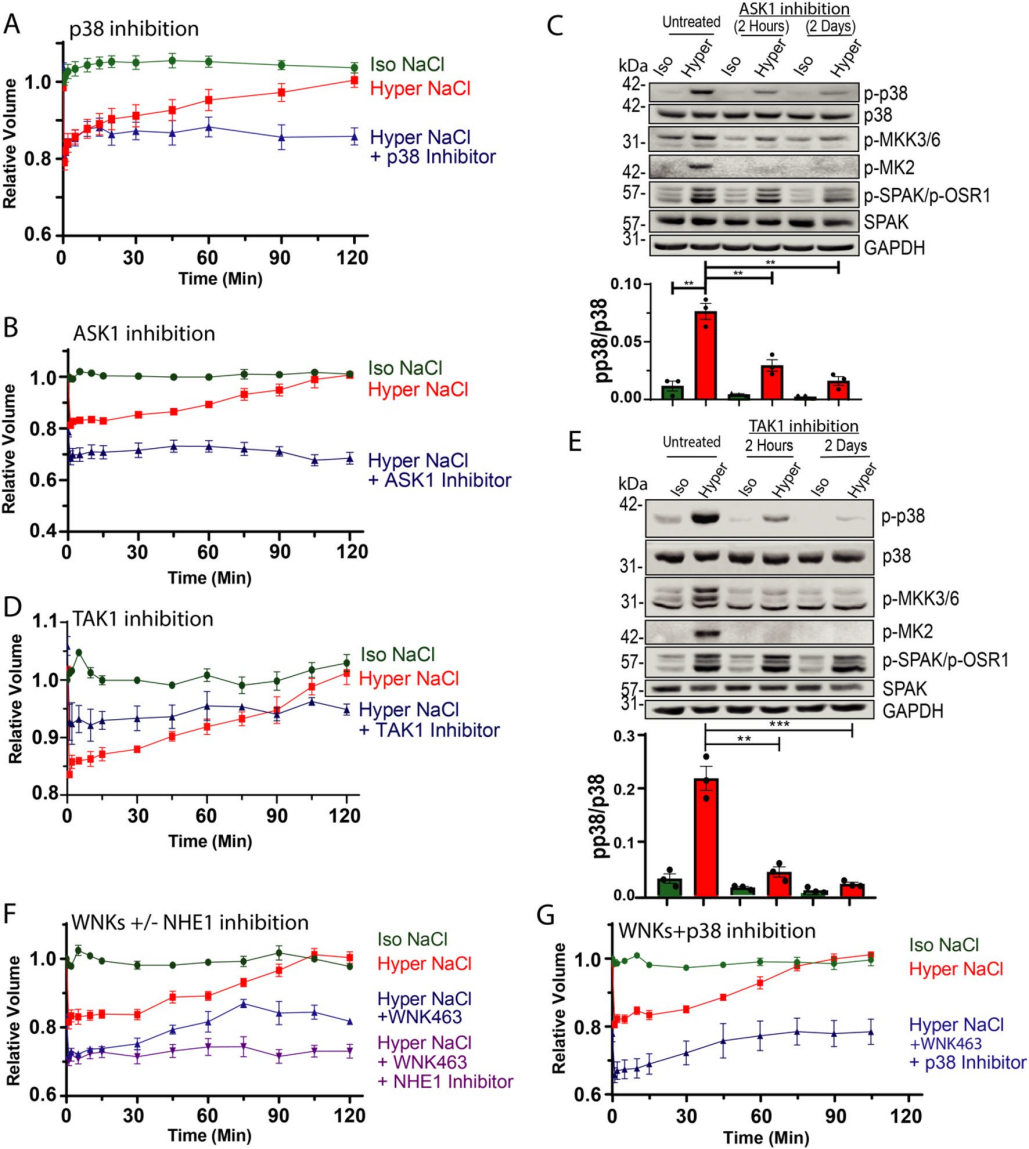
Long term p38 or WNKs inhibition (+ /− NHE1 inhibition) attenuates RVI. HeLa cells expressing scramble control plasmid were treated with (**A**) 5 μM SB203580 (p38 inhibitor), (**B**) 10 μM GS-444217 (ASK1 inhibitor), or (**D**) 1 μM 5Z-7-Oxozeaenol (TAK1 inhibitor) for 2 days, washed with PBS and exposed to iso- or hyper- osmotic NaCl solution in the presence of the inhibitors for the indicated times. Cell volume recovery (RVI) was measured by using a Coulter Counter. HeLa scramble control cells were treated with (**C**) 10 µM GS-444217 or (**E**) 1 μM 5Z-7-Oxozeaenol for 2 h or 2 days followed by incubation of iso- or hyper- osmotic NaCl solutions in the presence/absence of GS-444217 or 5Z-7-Oxozeaenol. Quantification of active p38 (p-p38) is depicted below their respective blots. (**F**) HeLa scramble control cells were treated with 10 μM WNK463 for 2 days with or without 30 min treatment of 10 μM Cariporide (NHE1 inhibitor). (**G**) HeLa scramble control cells were treated with 5 μM SB203580 and 10 μM WNK463 for 2 days. (**F, G**) After treatment with the indicated inhibitors, cells were washed with PBS and exposed to iso- or hyper- osmotic NaCl solution in the presence of the indicated inhibitors for the indicated times. RVI was measured as above. All data are mean ± s.e.m, N ≥ 3 independent experiments. Statistics as described in Fig. 1.

For the RVI experiments, HeLa cells were incubated in hyperosmotic solution, which led to an immediate cell shrinkage, as expected (see Figure S2 for cell shrinkage during the first 5 min. of hyperosmotic stress in all RVI experiments). The ensuing volume recovery took ~ 2 h to complete (Fig. 5A). Similar RVI dynamics were also seen in HeLa cells pre-incubated in hypo-osmotic solution, which normally triggers Regulatory Volume Decrease (RVD), prior to switching to hyperosmotic solution (Figure S3A). The RVI recovery was not blocked by short term (2 h) preincubation with the p38 inhibitor SB203580 (Figure S3B). However, long term (2 day) preincubation of the cells (prior to the hyperosmotic treatment) with the p38 inhibitor led to inhibition of RVI (Fig. 5A). In accord, 2-day (but not 2 h) incubation with an inhibitor of ASK1 (GS-444217), or TAK1 (5Z-7-Oxozeaenol), upstream activators of p38 (Fig. 3D), also suppressed RVI (Fig. 5B–E and Figures S2C-F and S3C,D). A 2-day, but not 2 h, incubation with the WNK inhibitor WNK463, attenuated RVI (Fig. 5F and Figure S3E). Unlike the effect of the pan WNK inhibitor (WNK463), stable knock-down (KD) of WNK1 alone did not affect RVI (Figure S3G). Long-term (2 day) inhibition of both p38 and WNKs together also attenuated RVI (Fig. 5G). These results suggest that WNKs and p38 may act (at least partially) in the same pathway to regulate RVI.

Since it is well-known that many cells utilize NHE1 transporters to regulate RVI^31^, we tested the effect of the NHE1 inhibitor, cariporide, on RVI in these HeLa cells. Our results show that while 30 min treatment with cariporide alone did not affect RVI (Figure S3F), its addition to the WNK inhibitor WNK463 completely abolished RVI (Fig. 5F), suggesting that NHE1 and WNKs operate in separate pathways to regulate RVI, and that blocking both pathways abolish RVI.

### NKCC1 promotes RVI via regulating p38

**Figure 6:**
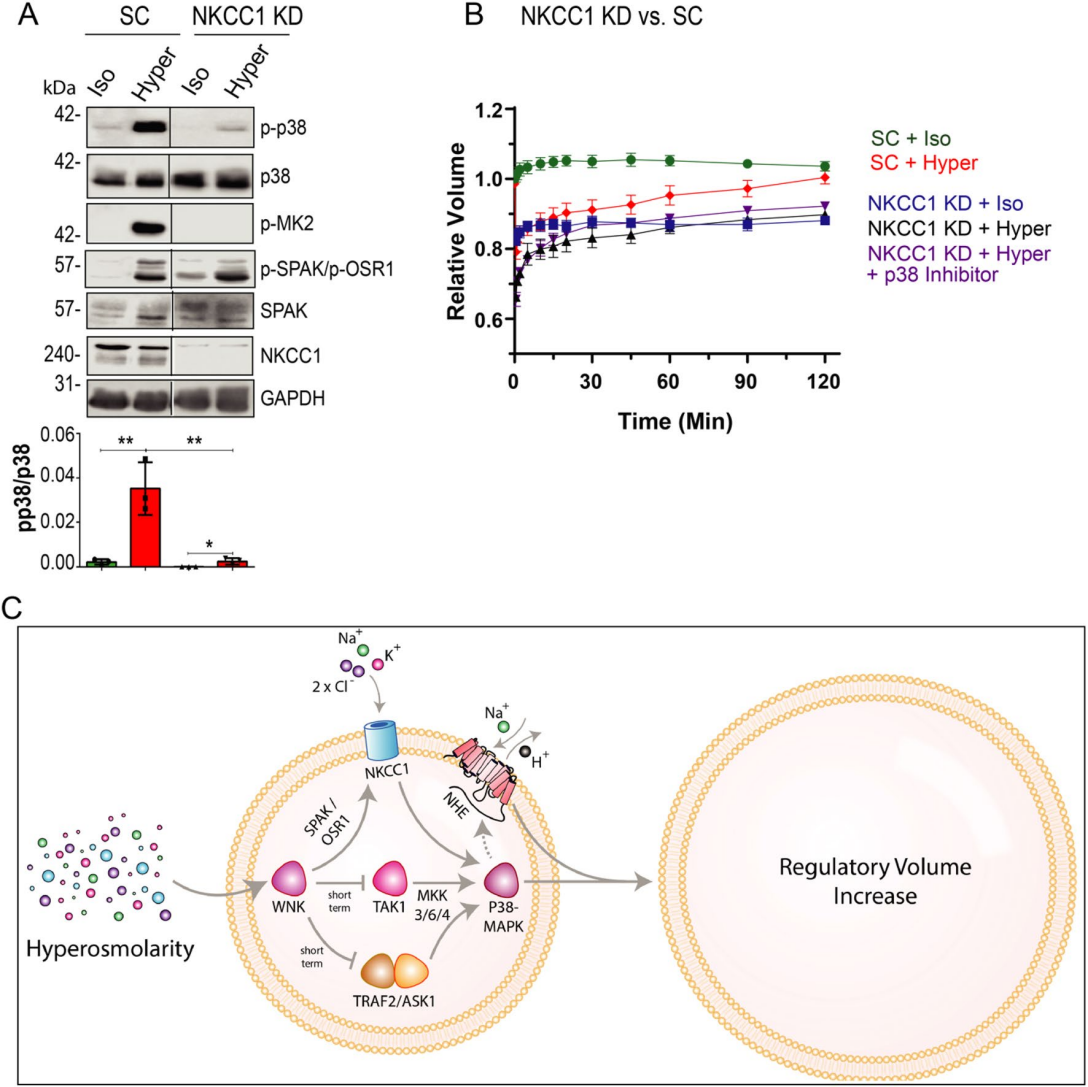
NKCC1 depletion inhibits p38 activation, and summary of pathways that regulate RVI. (**A**) NKCC1 stable knockdown (or scramble control, SC) HeLa cells were treated with iso- or hyper- osmotic NaCl solutions for 15 min. Quantification of active p38 (p-p38) is depicted below their respective blots. Black lines separate the left and right lanes of the same blot. (**B**) Scramble control (SC) or NKCC1 stable knockdown (KD) HeLa cells were treated (or not) with 5 μM SB203580 (p38 inhibitor) for 2 days, washed, exposed to iso- or hyper- osmotic NaCl solution for the indicated times, and RVI measured as described in Fig. 5. All data are mean ± s.e.m, *N* = 3 independent experiments. Statistics as described in Fig. 1. (**C**) A model depicting the regulation of RVI by the WNK- > NKCC1- > p38-MAPK pathway and by NHE1.

We recently showed that the ion transporter NKCC1, a known target of WNKs^32^, and well-known to promote RVI^5,14,33^, positively regulates p38 activity^14^; Indeed, stable knockdown of NKCC1 in HeLa cells led to a strong inhibition of p38 activity (Fig. 6A). Interestingly, while NKCC1-KD cells exhibited impaired RVI, as demonstrated by slower RVI kinetics (Figure S2L), inhibition of p38 in these cells exhibited identical attenuated RVI with no additive effect (Fig. 6B); this suggests that NKCC1 normally regulates RVI via activating p38.

Collectively, these results, and the known stimulatory effect of WNK on NKCC1^32^, suggest a WNK—> NKCC1—> p38 pathway that promotes RVI, along with a second stimulatory pathway mediated by NHE1 (Fig. 6C).

### Regulation of cell proliferation by p38 and WNK

**Figure 7:**
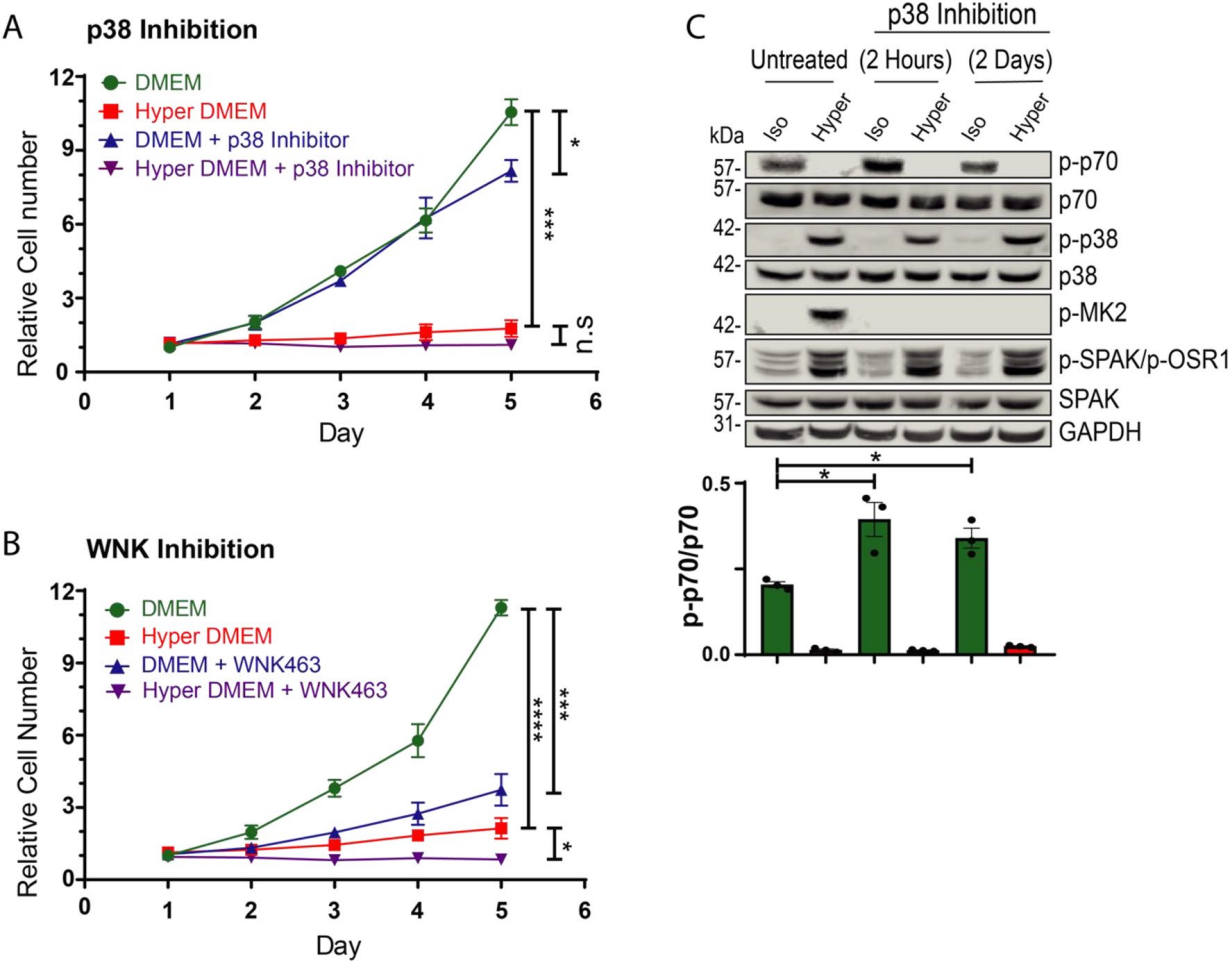
The effect of p38, WNKs and hyper-osmolarity on cell proliferation and mTORC1 activation. HeLa cells were treated with either iso- or hyper- osmotic cellular medium with or without (**A**) 5 μM SB203580 (p38 inhibitor), or (**B**) 10 μM WNK463 (pan-WNK inhibitor). Cell proliferation (cell count) was measured over the course of 5 days using a Coulter Counter, and normalized to Day 1. (**C**) HeLa cells were treated with 5 μM SB203580 for 2 h or 2 days and followed by incubation with iso- or hyper- osmotic DMEM (+ serum) for 15 min. in the presence/absence of SB203580. Quantification of active p70 S6K (p-p70) is depicted below the respective blots. All data are mean ± s.e.m, (*N *= 3 independent experiments). Statistics as described in Fig. 1.

Earlier work showed that p38 negatively regulates cell cycle progression, and coordinates cell cycle/cell proliferation with cell size, to ensure that only cells that reach a minimum size can enter the cell cycle^4^. Our recent work also showed that NKCC1 connects between cell volume/size and cell proliferation by regulating mTORC1^14^. We thus investigated the role of WNK and/or p38 in cell proliferation under iso-osmotic condition or hyperosmotic stress. As seen in Fig. 7A,B, hyperosmotic stress alone severely reduced cell proliferation. Under iso-osmotic conditions, p38 inhibition had only a small negative effect on cell proliferation (Fig. 7A), while WNK inhibition significantly reduced it (Fig. 7B). The combination of hyperosmolarity and either p38 or WNK inhibition abolished cell proliferation altogether (Fig. 7A,B). Interestingly, mTORC1 activation (p-p70) was blocked by hyperosmotic stress, as expected. Since p38 (which is stimulated by hyperosmolarity) inhibited mTORC1 activation (Fig. 7C) we tested whether p38 inhibition could prevent suppression of mTORC1. Our results show that while p38 inhibition indeed stimulated mTORC1 activity under iso-osmotic conditions, hyperosmotic stress blocked mTORC1 activation even in the presence of p38 inhibitors (Fig. 7C). This suggests that the hyperosmotic stress—dependent inhibition of mTORC1 is not mediated by p38. Thus, the inability of the p38 inhibitor to block cells proliferation under iso-osmotic conditions could be associated with the observed strong mTORC1 activity. Taken together, our results suggest that WNKs normally promotes cell proliferation, while p38 may attenuate it by inhibiting mTORC1; Hyperosmolarity suppresses cell proliferation under all conditions.

## Discussion

We show here that hyperosmolarity activates the p38 signaling cascade via stimulation of the TRAF2/ASK1-MKK3/MKK6/MKK4-p38-MK2 stress response pathways (Fig. 3D). We also show that a short term (2 h) inhibition of the osmo-sensors WNK kinases stimulate p38 activity, likely by stimulating TAK1 and ASK1/TRAF2; In contrast, a long-term (2 days) inhibition of WNKs with a pan-WNKs inhibitor, WNK463, suppresses p38. Long-term blockade of either p38 or WNK suppresses RVI and cell proliferation in cells (see model—Fig. 6C).

The activation of p38 by hyperosmolarity has been well studied in the past decades^6,9^. However, the most upstream osmo-sensor in the p38 pathway in our cells still remains unclear. Previous work by G. Johnson’s group showed that Rac1 is important in activating the p38 pathway in COS7 cells under hyperosmotic stress. They also discovered a scaffold protein, osmosensing scaffold for MEKK3 (OSM), and proposed that hypertonicity induces p38 via Rac1–OSM–MEKK3–MKK3–p38 pathway^24^. However, work from another group demonstrates an opposite result: Knockdown of Rac1 or OSM by siRNA increases p38 phosphorylation rather than decreases it^34^. Our own work also showed that inhibiting Rac1 with the inhibitor NSC23766 did not abolish p38 activation in hyperosmotic solutions (a higher dose of NSC23766 actually increased p38 activation). Thus, it is not clear under what conditions, or cell types, is Rac1 an upstream osmo-sensor of p38.

Instead, we focused on other upstream activators of p38. Our result demonstrated that the interaction between TRAF2 and ASK1 (MAP3K5) is important for activating the p38 pathway in cells exposed to hyperosmolarity. We also investigated if TNFR1, a known upstream activator of the TRAF2/ASK1 complex^35,36^, is the osmo-sensor for p38 activation. Surprisingly, in our studies TNFR1 depletion induced rather than inhibited p38 activation under hyperosmotic stress. Thus, the exact mechanism that promotes TRAF2/ASK1 complex formation induced by hyperosmolarity in our cells is so far unknown.

We also demonstrate that TAK1 (MAP3K7) is another important MAP3K that induces p38 activation in cells under hyperosmotic stress, although it is not yet known how TAK1 is activated by hyperosmolarity. In addition, we show, unexpectedly, that a short-term (2 h) treatment with WNK463 leads to p38 hyperactivation by inducing TAK1 phosphorylation, as well as by promoting ASK1/TRAF2 association, under hyperosmotic stress. This suggests that normally WNKs can inhibit p38 via suppressing TAK1 or ASK1/TRAF2 binding. However, in cells treated for 2 days with WNK463, TAK1 and p38 activation were reduced to levels similar to the untreated cells. MK2, the downstream target kinase of p38, was actually suppressed upon such long-term WNK463 treatment. The reason behind this observation is still unclear, but may represent a compensatory effect involving genomic or protein synthesis effects (see below). However, we cannot preclude the possibility that a long term WNK463 treatment affects other proteins aside from WNKs, which can, in turn, affect TAK1, p38 and/or MK2 activity. Of interest, in colonic organoids, WNK inhibition alone (even in the absence of hyperosmolarity) was sufficient to enhance MKK3/6 and p38 activation, revealing an exquisite sensitivity of p38 signaling to WNK in these cells. Currently, we do not know the biochemical pathway(s) involved, although it/they are clearly independent of SPAK/OSR1.

In a recent study (https://www.biorxiv.org/content/10.1101/2022.01.10.475707v1) it was demonstrated that hyperosmotic stress leads to sequestration of WNK1&3 in membrane-less droplets due to molecular crowding by the reduced cell volume; this promotes signaling that activates NKCC1, to induce RVI. An obvious question is how under these conditions is WNK1 (or WNK3?) able to inhibit p38? Is it via activation of signaling involving pSPAK/pOSR1 that then activate NKCC1? Or is p38 also clustered in liquid droplets along with the WNK. Future experiments will address these questions.

In our RVI studies, we show that a short-term treatment of cells with WNK463 or a p38 inhibitor (2 h) has no effect on RVI regulation. However, a long-term inhibition (2 days) of p38 (and its upstream kinases, ASK1 or TAK1) or WNKs, attenuate RVI. This result suggests that WNKs and p38-mediated regulation of RVI requires other downstream components, and possibly also genomic effects that lead to enhanced transcription of key genes and/or increased translation of critical proteins, yet to be identified.

We also show that NKCC1, known to be activated by WNKs^37^, is an activator of p38, suggesting a WNK- > NKCC1- > p38 pathway that promotes RVI in cells. However, we noted that WNKs inhibition did not lead to complete blockade of RVI. A possible explanation for this observation could be that p38 activation was not completely blocked by the long-term WNKs inhibition. In addition, earlier studies indicated that NHE1 activity can be induced by p38^38–40^. Thus, it is possible that a long-term WNK463 treatment, in which p38 is still active in our cells (albeit to a lesser extent), promotes NHE1 activity, allowing partial RVI to proceed. Moreover, the short-term WNK463 treatment, which failed to inhibit RVI, may also be explained by an increase of NHE1 activity induced by the hyperactivated p38. Thus, in order to completely block RVI in our cells, both the WNKs and NHE1 have to be inhibited together (Fig. 6C).

The observation of the involvement of p38 in RVI downstream of NKCC1 is curious, as active NKCC1 itself causes influx of the ions (followed by water) needed to restore cell volume. We recently showed that NKCC1 inhibits mTORC1 by blocking LAT1 and the Akt/Erk pathways (which activate mTORC1), and by promoting p38 (which inhibits mTORC1)^14^. Thus, it is possible that mTORC1 inhibition and the ensuing blockade of protein synthesis leads to the generation of steady-state smaller cell size, as indeed we observed here (Fig. 5A), and as we previously reported for NKCCI-KD cells^14^; This thus enables recovery to a new steady-state smaller cell volume.

A recent study showed that NKCC1-dependent RVI was impaired in WNK1 knockout HEK293 cells, especially when treated also with a NHE1 inhibitor (^16^, and Subramanya, unpublished). Since we showed that in our own generated stable HeLa WNK1-KD cells p38 activity is elevated, the obvious question is why a long-term (2 day) treatment of cells with WNK463 leads to a reduction in p38 activation in our current study, while WNK1 knockout or knockdown results in elevated p38 activity. It is possible that long-term treatment with WNK463 has a strong effect on the other WNKs (e.g. WNK 3—known to be sensitive to hypertonic stress and involved in RVI^41^), which in-turn exert their effects on p38. Lastly, we cannot preclude the possibility that a long-term WNK463 treatment may also affect other proteins in addition to WNKs.

Our work also shows that hyperosmotic stress strongly decreases cell proliferation rate, consistent with other studies^42,43^ and in line with our observed strong inhibition of mTORC1 under hyperosmolarity. We also demonstrate that WNK kinases are important for promoting cell proliferation, as treatment with WNK463 significantly attenuated it in iso-osmotic conditions, and completely blocked it under hyper-osmotic stress. In contrast, p38 inhibition in iso-osmotic medium had only a small (and delayed) effect on inhibiting cell proliferation, only observed after 5 days. It is thus possible that the sustained mTORC1 activation following p38 inhibition is responsible for the almost normal, un-attenuated cell proliferation in our experiments. In agreement, in our previous study we demonstrated an increase of cell division rate/cell proliferation when NKCC1 was depleted^14^. Since NKCC1 depletion suppresses p38 activation, similar to p38 inhibition with a drug, this suppression promotes mTORC1 activation, as we show here and in our recent paper^14^; this, in turn, induces cell proliferation.

Taken together, our current study demonstrates that there is a tight connection between the WNK and the p38 pathways, with WNKs regulating the p38 pathway. Both pathways are important in regulating volume recovery (RVI) and cell proliferation.

## Materials and methods

Reagents and solution: see Tables S1 and Table S2 in the Supplementary Material.

### Methods

#### Generation of TNFRα (TNFR1), ASK1 and WNK1 knock-down (KD) cells

HeLa cells were transfected with human TNFR1 shRNA construct (V2LHS 94,071, Dharmacon), ASK1 shRNA construct (V2LHS_198510, Dharmacon) and WNK1 shRNA construct (V3LHS_638999, Dharmacon). The cells were then selected with Puromycin (2 µg/ml) 24 h after transfection. Medium was changed every 2–3 days. Once the transfected cells were established and expanded, TNFRα, ASK1, or WNK1 knockdown was verified by immunoblotting.

#### Immunoblotting

HeLa, HEK293, MDCK, human primary bronchial epithelial cells, or mouse colonic organoids (generated as described in^14^), were washed in PBS, and treated with either iso-osmotic or hyperosmotic solutions (Table S2) for 15 min at 37 °C, and processed as indicated. For a short-term inhibitor treatment, cells were treated with either 10 μM WNK463 (a pan-WNK-kinase inhibitor), 5 µM SB203580 (p38 inhibitor), or 10 µM GS-444217 (ASK1 inhibitor) for 2 h, or 1 µM 5Z-7-Oxozeaenol (TAK1 inhibitor) for 1 h prior to (and during) incubation with the iso- or hyper- osmotic solutions. For a long-term inhibitor treatment, cells were treated with the indicated inhibitor for 2 days prior to (and during) incubation with the iso- or hyper- osmotic solutions. Cells were then lysed in lysis buffer (50 mM Hepes, pH 7.5, 150 mM NaCl, 1% Triton X-100, 10% glycerol, 1.5 mM MgCl_2_, 1.0 mM EGTA, and 10 μg/ml of each leupeptin, aprotinin and pepstatin, and 1 mM phenylmethanesulfonylfluoride (PMSF)). Proteins were then separated on SDS-PAGE and immunoblotted with the indicated antibodies. All immunoblots were imaged using the Odyssey Imaging system (Odssey Fc, LI-COR) and quantified using Image Studio version 5.2 (LI-COR).

#### Co-Immunoprecipitation (Co-IP) assays

HeLa cells were transfected with the specified cDNA constructs for two days prior to any treatment. Then, cells were treated with either iso-osmotic or hyperosmotic NaCl solutions for 15 min and lysed in lysis buffer. Co-IP of ASK1-FLAG and HA-TRAF2 were analyzed by IP of ASK1-FLAG from 1 mg of cleared cell lysate with anti-FLAG M2 affinity beads and immunoblotting with either anti-HA, anti-FLAG, or other specified antibodies. Blots were imaged as above.

#### Regulatory volume increase (RVI) experiments

HeLa (scramble control (SC), WNK1 knockdown (KD) or NKCC1 KD) cells were grown in 6 well tissue culture plates. Cells were dissociated using trypsin and treated with either iso-osmotic or hyperosmotic NaCl solutions (Table S2) for the indicated times. The starting cell volume for each experiment was the cell volume at time 0 of the control, untreated cells. For drug inhibition experiments, cells were treated with either 5 µM SB203580 (p38 inhibitor), 10 µM WNK463 (pan-WNKs inhibitor), 10 µM GS-444217 (ASK1 inhibitor), or 1 µM 5Z-7-Oxozeaenol (TAK1 inhibitor), for 2 h (short-term) or 2 days (long term) prior to RVI analysis. Cell diameter was measured using a Multisizer 4 Coulter Counter (Beckman-Coulter).

#### Measurements of intracellular Na^+^ and K^+^ concentrations

For intracellular Na^+^ ([Na^+^]_i_) and K^+^ ([K^+^]_i_) measurements, cells were grown in 10 cm tissue culture plates and treated with the various osmotic solutions (Table S2) for 15 min. They were then quickly washed 3 times with osmotically balanced solution of 150 mM LiCl (300 mOsm) on ice. The cells were then lysed overnight at 4 °C in 1% HNO_3_. The [Na^+^]_i_ and [K^+^]_i_ were measured using a PinAAcle 900F Atomic Absorption Spectrometer (PerkinElmer) and then normalized to cell number and cell volume.

#### Cell proliferation assay

HeLa cells were seeded at 10^4^ cells per well in a 24-well plate. After the measurement of cell number on day 1, hyperosmotic cellular medium (Table S2) and 5 µM of SB203850 or 10 µM of WNK463 were added where indicated. Cell proliferation was determined by counting cells at the indicated times using the Multisizer 4 Coulter Counter (Beckman-Coulter) and normalizing to day 1.

#### Quantification and statistical analysis

All immunoblots were imaged using the Odyssey Imaging system and quantified using Image Studio version 5.2 (LI-COR). Each experiment was performed for at least three independent times. Statistic analysis was performed by using GraphPad Prism 8. Histogram bars represent mean ± s.e.m. *p*-values < 0.05 were considered statistically significant and indicated in the Figures (*p* values: Not significant (n.s) > 0.05, * < 0.05, ** < 0.01, *** < 0.001, **** < 0.0001, using unpaired student *t*-tests).

## Supplementary Information

Supplementary Information.

## Data Availability

All data generated or analysed during this study are included in this published article [and its supplementary information files].

## References

[1] Pearson G (2001). Mitogen-activated protein (MAP) kinase pathways: Regulation and physiological functions. Endocr. Rev..

[2] Thornton TM, Rincon M (2009). Non-classical p38 map kinase functions: Cell cycle checkpoints and survival. Int. J. Biol. Sci..

[3] Cuenda A, Rousseau S (2007). p38 MAP-kinases pathway regulation, function and role in human diseases. Biochim. Biophys. Acta.

[4] Liu S (2018). Size uniformity of animal cells is actively maintained by a p38 MAPK-dependent regulation of G1-length. Elife.

[5] Arroyo JP, Kahle KT, Gamba G (2013). The SLC12 family of electroneutral cation-coupled chloride cotransporters. Mol. Asp. Med..

[6] Hoffmann EK, Lambert IH, Pedersen SF (2009). Physiology of cell volume regulation in vertebrates. Physiol. Rev..

[7] Grinstein S, Clarke CA, Rothstein A (1983). Activation of Na+/H+ exchange in lymphocytes by osmotically induced volume changes and by cytoplasmic acidification. J. Gen. Physiol..

[8] Gamba G (2005). Molecular physiology and pathophysiology of electroneutral cation-chloride cotransporters. Physiol. Rev..

[9] Han J, Lee JD, Bibbs L, Ulevitch RJ (1994). A MAP kinase targeted by endotoxin and hyperosmolarity in mammalian cells. Science.

[10] Hoffmann EK, Pedersen SF (2007). Shrinkage insensitivity of NKCC1 in myosin II-depleted cytoplasts from Ehrlich ascites tumor cells. Am. J. Physiol. Cell Physiol..

[11] Moriguchi T (1996). Purification and identification of a major activator for p38 from osmotically shocked cells. Activation of mitogen-activated protein kinase kinase 6 by osmotic shock, tumor necrosis factor-alpha, and H_2_O_2_. J. Biol. Chem..

[12] Roger F, Martin PY, Rousselot M, Favre H, Feraille E (1999). Cell shrinkage triggers the activation of mitogen-activated protein kinases by hypertonicity in the rat kidney medullary thick ascending limb of the Henle's loop. Requirement of p38 kinase for the regulatory volume increase response. J. Biol. Chem..

[13] Bildin VN, Wang Z, Iserovich P, Reinach PS (2003). Hypertonicity-induced p38MAPK activation elicits recovery of corneal epithelial cell volume and layer integrity. J. Membr. Biol..

[14] Demian WL (2019). The ion transporter NKCC1 links cell volume to cell mass regulation by suppressing mTORC1. Cell Rep..

[15] Gamba G (2005). Role of WNK kinases in regulating tubular salt and potassium transport and in the development of hypertension. Am. J. Physiol. Renal. Physiol..

[16] Roy A (2015). Generation of WNK1 knockout cell lines by CRISPR/Cas-mediated genome editing. Am. J. Physiol. Renal. Physiol..

[17] Murillo-de-Ozores AR, Chavez-Canales M, de Los Heros P, Gamba G, Castaneda-Bueno M (2020). Physiological processes modulated by the chloride-sensitive WNK-SPAK/OSR1 kinase signaling pathway and the cation-coupled chloride cotransporters. Front. Physiol..

[18] Derijard B (1995). Independent human MAP-kinase signal transduction pathways defined by MEK and MKK isoforms. Science.

[19] Stein B, Brady H, Yang MX, Young DB, Barbosa MS (1996). Cloning and characterization of MEK6, a novel member of the mitogen-activated protein kinase kinase cascade. J. Biol. Chem..

[20] Meier R, Rouse J, Cuenda A, Nebreda AR, Cohen P (1996). Cellular stresses and cytokines activate multiple mitogen-activated-protein kinase kinase homologues in PC12 and KB cells. Eur. J. Biochem..

[21] Risco A, Cuenda A (2012). New insights into the p38gamma and p38delta MAPK pathways. J. Signal Transduct..

[22] Nishitoh H (1998). ASK1 is essential for JNK/SAPK activation by TRAF2. Mol. Cell.

[23] Amos LA (2018). ASK1 inhibitor treatment suppresses p38/JNK signalling with reduced kidney inflammation and fibrosis in rat crescentic glomerulonephritis. J. Cell Mol. Med..

[24] Uhlik MT (2003). Rac-MEKK3-MKK3 scaffolding for p38 MAPK activation during hyperosmotic shock. Nat. Cell Biol..

[25] Bradley JR, Pober JS (2001). Tumor necrosis factor receptor-associated factors (TRAFs). Oncogene.

[26] de Los Heros P, Pacheco-Alvarez D, Gamba G (2018). Role of WNK kinases in the modulation of cell volume. Curr. Top Membr..

[27] Yamada K (2016). Small-molecule WNK inhibition regulates cardiovascular and renal function. Nat. Chem. Biol..

[28] Choo MK, Sakurai H, Koizumi K, Saiki I (2006). TAK1-mediated stress signaling pathways are essential for TNF-alpha-promoted pulmonary metastasis of murine colon cancer cells. Int. J. Cancer.

[29] Arai Y (2020). WNK1-TAK1 signaling suppresses lipopolysaccharide-induced cytokine production and classical activation in macrophages. Biochem. Biophys. Res. Commun..

[30] Ninomiya-Tsuji J (2003). A resorcylic acid lactone, 5Z-7-oxozeaenol, prevents inflammation by inhibiting the catalytic activity of TAK1 MAPK kinase kinase. J. Biol. Chem..

[31] Rotin D, Grinstein S (1989). Impaired cell volume regulation in Na(+)-H+ exchange-deficient mutants. Am. J. Physiol..

[32] Gagnon KB, England R, Delpire E (2006). Characterization of SPAK and OSR1, regulatory kinases of the Na-K-2Cl cotransporter. Mol. Cell Biol..

[33] Walcott B, Birzgalis A, Moore LC, Brink PR (2005). Fluid secretion and the Na+-K+-2Cl- cotransporter in mouse exorbital lacrimal gland. Am. J. Physiol. Cell Physiol..

[34] Zhou X, Izumi Y, Burg MB, Ferraris JD (2011). Rac1/osmosensing scaffold for MEKK3 contributes via phospholipase C-gamma1 to activation of the osmoprotective transcription factor NFAT5. Proc. Natl. Acad. Sci. USA.

[35] Liu H, Nishitoh H, Ichijo H, Kyriakis JM (2000). Activation of apoptosis signal-regulating kinase 1 (ASK1) by tumor necrosis factor receptor-associated factor 2 requires prior dissociation of the ASK1 inhibitor thioredoxin. Mol. Cell. Biol..

[36] Al-Lamki RS (2005). TNFR1- and TNFR2-mediated signaling pathways in human kidney are cell type-specific and differentially contribute to renal injury. FASEB J..

[37] Delpire E, Gagnon KB (2008). SPAK and OSR1: STE20 kinases involved in the regulation of ion homoeostasis and volume control in mammalian cells. Biochem. J..

[38] Khaled AR (2001). Trophic factor withdrawal: p38 mitogen-activated protein kinase activates NHE1, which induces intracellular alkalinization. Mol. Cell Biol..

[39] Pederson SF, Varming C, Christensen ST, Hoffmann EK (2002). Mechanisms of activation of NHE by cell shrinkage and by calyculin A in Ehrlich ascites tumor cells. J. Membr. Biol..

[40] Fang Z (2020). Burn-induced apoptosis of pulmonary microvascular endothelial cell is NHE1 dependent and regulated by PI3K-Akt and p38 MAPK pathways. Shock.

[41] Pacheco-Alvarez D (2020). WNK3 and WNK4 exhibit opposite sensitivity with respect to cell volume and intracellular chloride concentration. Am. J. Physiol. Cell Physiol..

[42] Li H, Wang J, Li F, Chen G, Chen Q (2018). The influence of hyperosmolarity in the intervertebral disc on the proliferation and chondrogenic differentiation of nucleus pulposus-derived mesenchymal stem cells. Cells Tissues Organs.

[43] Grauso M, Lan A, Andriamihaja M, Bouillaud F, Blachier F (2019). Hyperosmolar environment and intestinal epithelial cells: Impact on mitochondrial oxygen consumption, proliferation, and barrier function in vitro. Sci. Rep..

