# Supplementary material for: Regulation of the p38-MAPK pathway by hyperosmolarity and by WNK kinases
Source: Sci Rep. 2022 Aug 25;12:14480. doi: 10.1038/s41598-022-18630-w (PMC9411163; doi:10.1038/s41598-022-18630-w)

SUPPLEMENTARY MATERIAL

Regulation of the p38-MAPK pathway by hyperosmolarity and by WNK kinases

Zetao Liu[1,2], Wael Demian[1,2], Avinash Persaud[1], Chong Jiang[1], Arohan R. Subramanaya[3], Daniela Rotin[1,2,*]

[1]Cell Biology Program, the Hospital for Sick Children, and [2]Biochemistry Department, University of Toronto, Canada, [3]Department of Medicine and Cell Biology, University of Pittsburgh, USA

Running title: p38-MAPK regulation by hyperosmolarity and by WNKs

*Correspondence: Dr. Daniela Rotin, The Hospital for Sick Children, Cell Biology Program, PGCRL 19-9715, 686 Bay St., Toronto, Ontario, Canada, M5G 0A4

SUPPLEMENTARY TABLES

Table S1. Key resources of chemical, reagents and antibodies

REAGENT or RESOURCE	SOURCE	IDENTIFIER
**Antibodies**		
**Mouse anti-SPAK (STK39)**	EMD Millipore	Cat# MABS178
**Mouse anti-β-actin**	Sigma-Aldrich	Cat# A2228; RRID:AB_47669
**Mouse anti-phospho-p38 MAPK (T180/Y182)**	BD Bioscience	Cat# 612280; RRID:AB_399597
**Mouse anti-vinculin**	Santa Cruz	Cat# sc-25336; RRID:AB_628438
**Rabbit anti-NKCC1**	Cell Signaling	Cat# 8351; RRID:AB_10830068
**Rabbit anti-phospho-SPAK antibody (Ser-373)/phospho-OSR1 antibody (Ser-325)**	EMD Millipore	Cat# 07-2273
**Rabbit anti-phospho-MKK3 (Ser189)/MKK6 (Ser207)**	Cell Signaling	Cat# 12280
**Rabbit anti-phospho-SEK1/MKK4 (Ser257/Thr261)**	Cell Signaling	Cat# 9156
**Rabbit anti-p38 MAPK**	Cell Signaling	Cat# 9212; AB_330713
**Rabbit anti-GAPDH**	Cell Signaling	Cat# 5174
**Rabbit anti-phospho-MAPKAPK-2 (p-MK2) (Thr334)**	Cell Signaling	Cat# 3007
**Rabbit anti-WNK1**	Cell Signaling	Cat# 4979
**Rabbit anti-phospho-TRAF2 (Ser11)**	Cell Signaling	Cat# 13908
**Rabbit anti-TRAF2**	Cell Signaling	Cat# 4724
**Rabbit anti-phospho-TAK1 (Ser412)**	Cell Signaling	Cat# 9339
**Rabbit anti-TAK1**	Cell Signaling	Cat# 5206
**Rabbit anti-phospho-p70 S6 Kinase (Thr389)**	Cell Signaling	Cat# 9205
**Mouse anti-p70 S6 Kinase**	Santa Cruz	Cat# SC-8418
**Mouse anti MKK3/MKK6**	R&D Systems	Cat# MAB2514
**Rabbit anti-MAPKAPK-2 (MK2)**	Cell Signaling	Cat# 3042
**Rabbit anti-TNFR1**	Proteintech	Cat# 21574-1-AP
**Rabbit anti-ASK1**	Cell Signaling	Cat# 3762
**Mouse anti-FLAG**	Sigma	Cat# F1804-5MG
**Chemicals, Peptides, Transfection reagents and Recombinant Proteins**		
**SB203580**	SelleckChem	Cat# S1076
**WNK463**	SelleckChem	Cat# S8358
**Puromycin**	Bioshop	Cat# PUR333
**GS-444217**	MedKoo Biosciences	Cat# 564706
**5Z-7-Oxozeaenol**	Tocris	Cat# 3604
**NSC23766**	Tocris	Cat# 2161
**Cariporide**	Tocris	Cat# 5358
**Advanced DMEM/F-12**	Thermo Fisher	Cat# 12634028
**Intesticult Organoid Growth Medium (mouse)**	Stem Cell	Cat# 06005
**Corning Matrigel**	Corning	Cat# 356231
**PolyJet In Vitro DNA Transfection Reagent**	SignaGen Laboratories	Cat# SL100688
**Lipofectamine 3000 Transfection Reagent**	Thermo Fisher	Cat# 3000015
**BLUelf Prestained Protein Ladder**	FroggoBio	Cat# PM008-0500
**Anti-FLAG M2 Affinity gel**	Sigma	Cat# A2220-5ML

**Plasmids**		
**pcDNA3.1-MAP3K5 (ASK1) WT**	Addgene	Plasmid#: 47104
**pcDNA3 HA-humanTRAF2**	Addgene	Plasmid#: 66932
**Experimental Models: Cell Lines**		
**HeLa**	ATCC	Cat#CCL-2
**HEK293**	ATCC	Cat#CRL-1573
**MDCK**	ATCC	Cat#CCL-34
**Human Primary Bronchial Epithelial Cells**	Abm	Cat# T4006
**Scramble Control knockdown (KD) HeLa cells**	(14)	
**NKCC1 knockdown (KD) HeLa cells**	(14)	
**WNK1 knockdown (KD) HeLa cells**	Rotin lab	This study
**TNFR1 knockdown (KD) HeLa cells**	Rotin lab	This study
**ASK1 knockdown (KD) HeLa cells**	Rotin lab	This study
**Recombinant DNA**		
**pGIPZ human TNFR1 shRNA**	Dharmacon	V2LHS 94071
**pGIPZ human WNK1 shRNA**	Dharmacon	V3LHS_638999
**pGIPZ scramble shRNA**	Dharmacon	RHS4348
**pGIPZ human MAP3K5 (ASK1) shRNA**	Dharmacon	V2LHS_198510
**Software and Algorithms**		
**Syngistix**	PerkinElmer	N/A
**Prism 8**	GraphPad	N/A
**Image StudioVersion 5.2**	Li-Cor	N/A
**Multisizer 4e**	BECKMAN COULTER	N/A

Table S2. Osmotic solutions used in western blot experiments, intracellular Na^+ or K^+ concentration measurements and cell proliferation assays.

Condition	Isosmotic	Hyperosmotic
**NaCl solutions**	**315 mOsm:**<br>135 mM NaCl, 2.5 mM KCl, 0.25 mM $CaCl_2$, 0.25 mM $MgCl_2$, 5 mM glucose, 15 mM Hepes, pH 7.4	**400 mOsm:**<br>180 mM NaCl, 3.33 mM KCl, 0.25 mM $CaCl_2$, 0.25 mM $MgCl_2$, 5 mM glucose, 15 mM Hepes, pH 7.4<br>**475 mOsm:**<br>225 mM NaCl, 4.16 mM KCl, 0.25 mM $CaCl_2$, 0.25 mM $MgCl_2$, 5 mM glucose, 15 mM Hepes, pH 7.4<br>**580 mOsm:**<br>270 mM NaCl, 5 mM KCl, 0.25 mM $CaCl_2$, 0.25 mM $MgCl_2$, 5 mM glucose, 15 mM Hepes, pH 7.4
**NMDG-Cl solutions**	**315 mOsm:**<br>135 mM NMDG-Cl, 2.5 mM KCl, 0.25 mM $CaCl_2$, 0.25 mM $MgCl_2$, 5 mM glucose, 15 mM Hepes, pH 7.4	**400 mOsm:**<br>180 mM NMDG-Cl, 3.33 mM KCl, 0.25 mM $CaCl_2$, 0.25 mM $MgCl_2$, 5 mM glucose, 15 mM Hepes, pH 7.4<br>**475 mOsm:**<br>225 mM NMDG-Cl, 4.16 mM KCl, 0.25 mM $CaCl_2$, 0.25 mM $MgCl_2$, 5 mM glucose, 15 mM Hepes, pH 7.4<br>**580 mOsm:**<br>270 mM NMDG-Cl, 5 mM KCl, 0.25 mM $CaCl_2$, 0.25 mM $MgCl_2$, 5 mM glucose, 15 mM Hepes, pH 7.4
**Na-Gluconate solutions**	**315 mOsm:**<br>135 mM Na-Gluconate, 2.5mM Kgluconate, 0.25 mM $CaCl_2$, 0.25 mM $MgCl_2$, 5 mM glucose, 15 mM Hepes, pH 7.4	**580 mOsm:**<br>270 mM Na-Gluconate, 5 mM K-gluconate, 0.25 mM $CaCl_2$, 0.25 mM $MgCl_2$, 5 mM glucose, 15 mM Hepes, pH 7.4
**Choline – Cl solutions**	**315 mOsm:**<br>135 mM Choline-Cl, 2.5 mM KCl, 0.25 mM $CaCl_2$, 0.25 mM $MgCl_2$, 5 mM glucose, 15 mM Hepes, pH 7.4	**580 mOsm:**<br>270 mM Choline-Cl, 5 mM KCl, 0.25 mM $CaCl_2$, 0.25 mM $MgCl_2$, 5 mM glucose, 15 mM Hepes, pH 7.4
**Cellular Medium**	**315 mOsm:**<br>DMEM + 10% FBS	**475 mOsm:**<br>DMEM + 10% FBS + 90 mM NaCl

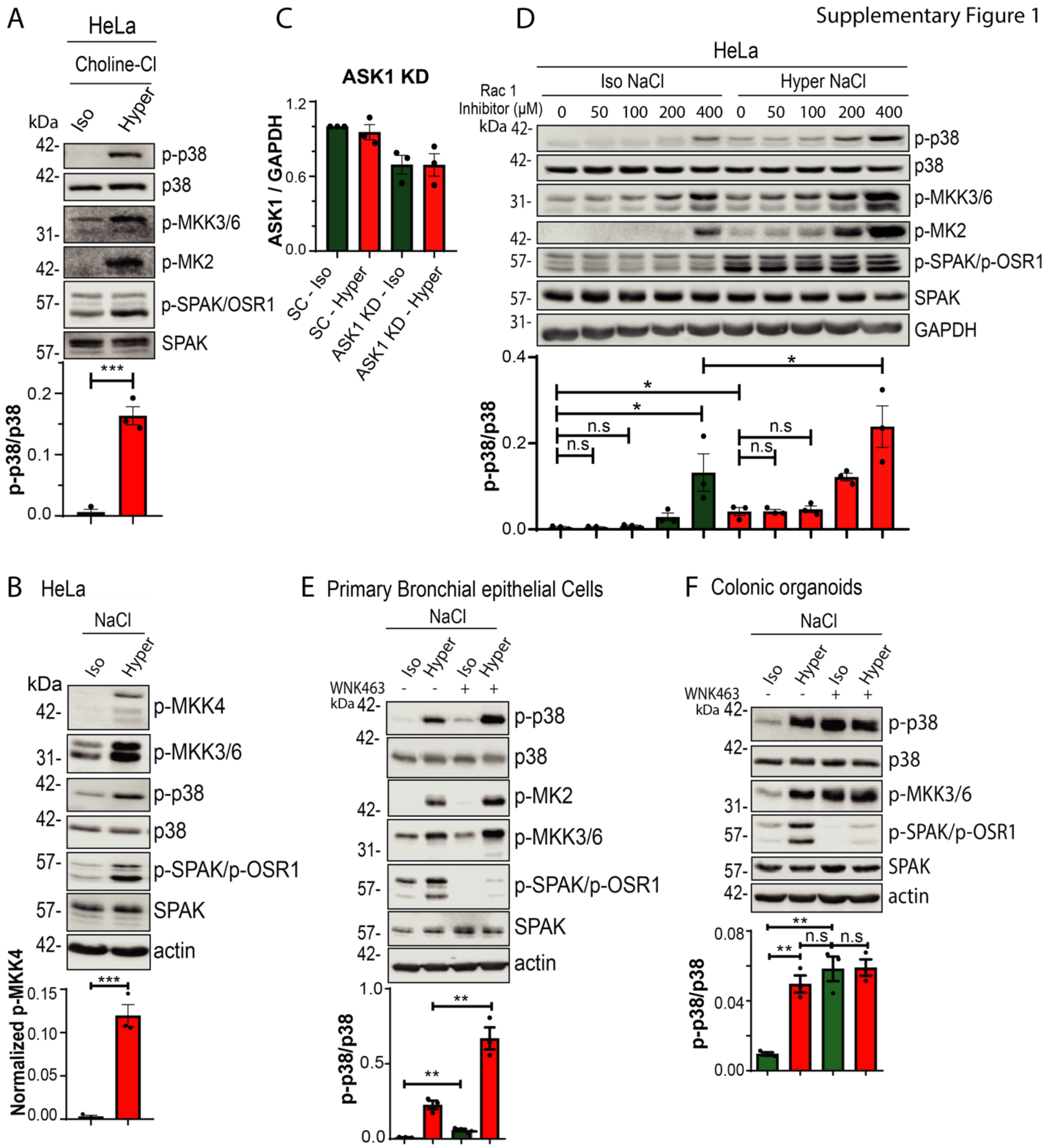

Supplementary Figure 1. Activation of the p38 pathway by hyperosmotic or hypertonic solutions, Rac1 Inhibitor or WNK463, in different cell types.

(A) HeLa cells were treated with Iso- or hyper- osmotic Choline-Cl solutions for 15 minutes. **(B)** HeLa cells were treated with iso-osmotic or hyper-osmotic NaCl solutions for 15 minutes. (**C**) Quantitation of ASK1 levels in ASK1-KD HeLa cells relative to scramble control (SC). **(D)** HeLa cells were treated with an increasing concentration of NSC23766 (Rac1 inhibitor) for 2 hours followed by incubation with either iso- or hyper-osmotic NaCl solution in the absence/presence of NSC23766 . **(E)** Human Primary

Bronchial Epithelial cells, or **(F)** Mouse Colonic Organoids, were treated with 10 µM WNK463 for 2 hours followed by incubation with iso- or hyper- osmotic NaCl for 15 minutes in the absence/presence of WNK463. Quantification of active p38 (p-p38) or MKK4 (p-MKK4) is depicted below their respective blots. All data are mean±s.e.m, N≥3 independent experiments. Statistics as described in Figure 1.

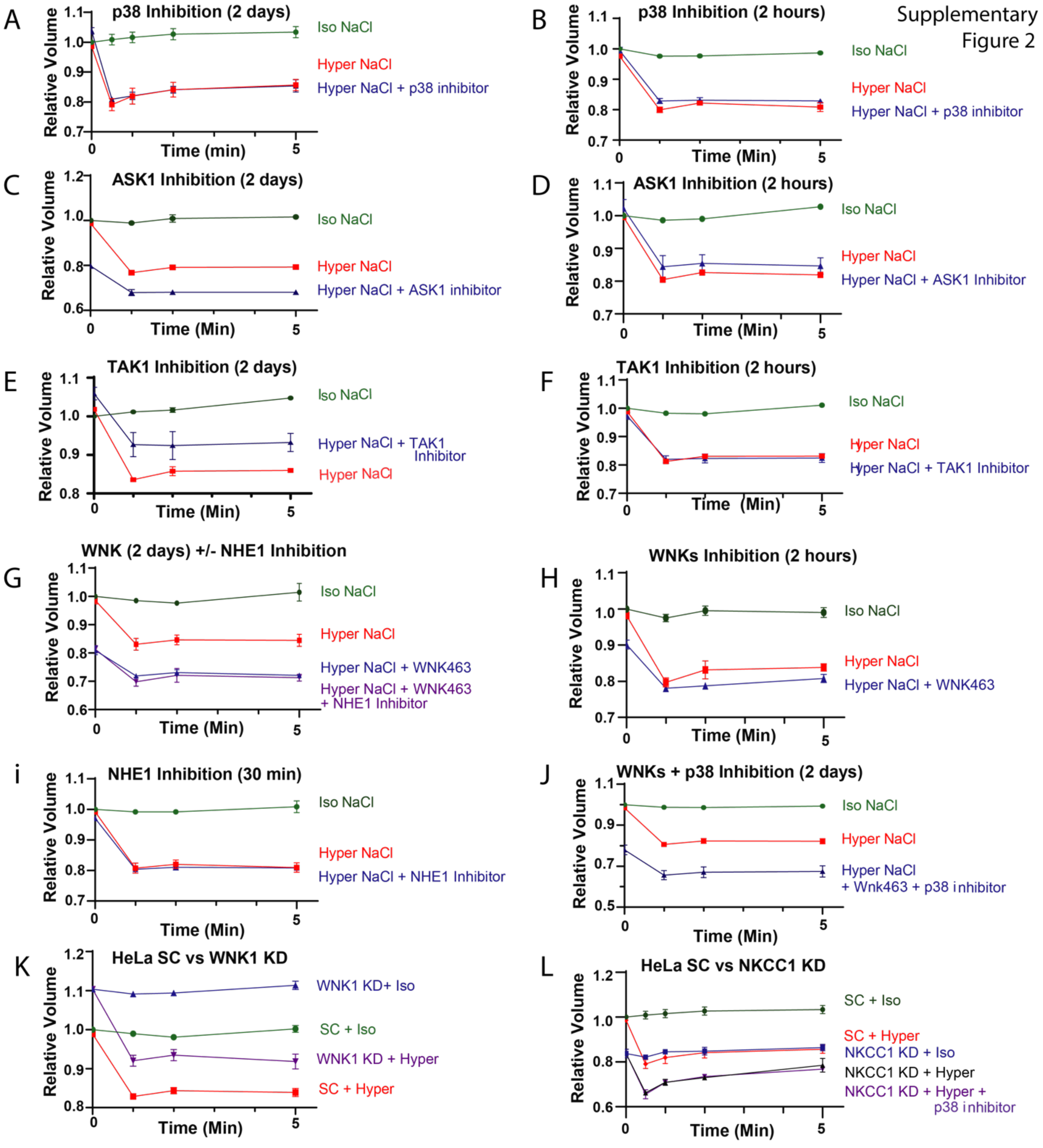

Supplementary Figure 2. Cell volume changes in the first 5 minutes of the RVI experiments.

Cell volume changes in the first 5 minutes of the respective RVI experiments shown in Figures 5 and 6. p38 inhibition for **(A)** 2 days or **(B)** 2 hours; ASK1 inhibition for **(C)** 2 days or **(D)** 2 hours; TAK1 inhibition for **(E)** 2 days or **(F)** 2 hours; **(G)** WNK inhibition with or without 30 minutes NHE1 inhibition; **(H)** WNKs inhibition for 2 hours; **(I)** NHE1 inhibition for 30 minutes; **(J)** WNKs and p38 inhibition for 2 days; **(K)** HeLa scramble control (SC) or WNK1 knockdown (KD) cells; **(L)** HeLa scramble control (SC) or NKCC1 KD cells. Cell volume was measured by using a Coulter counter. All data are mean±s.e.m, N≥3 independent experiments.

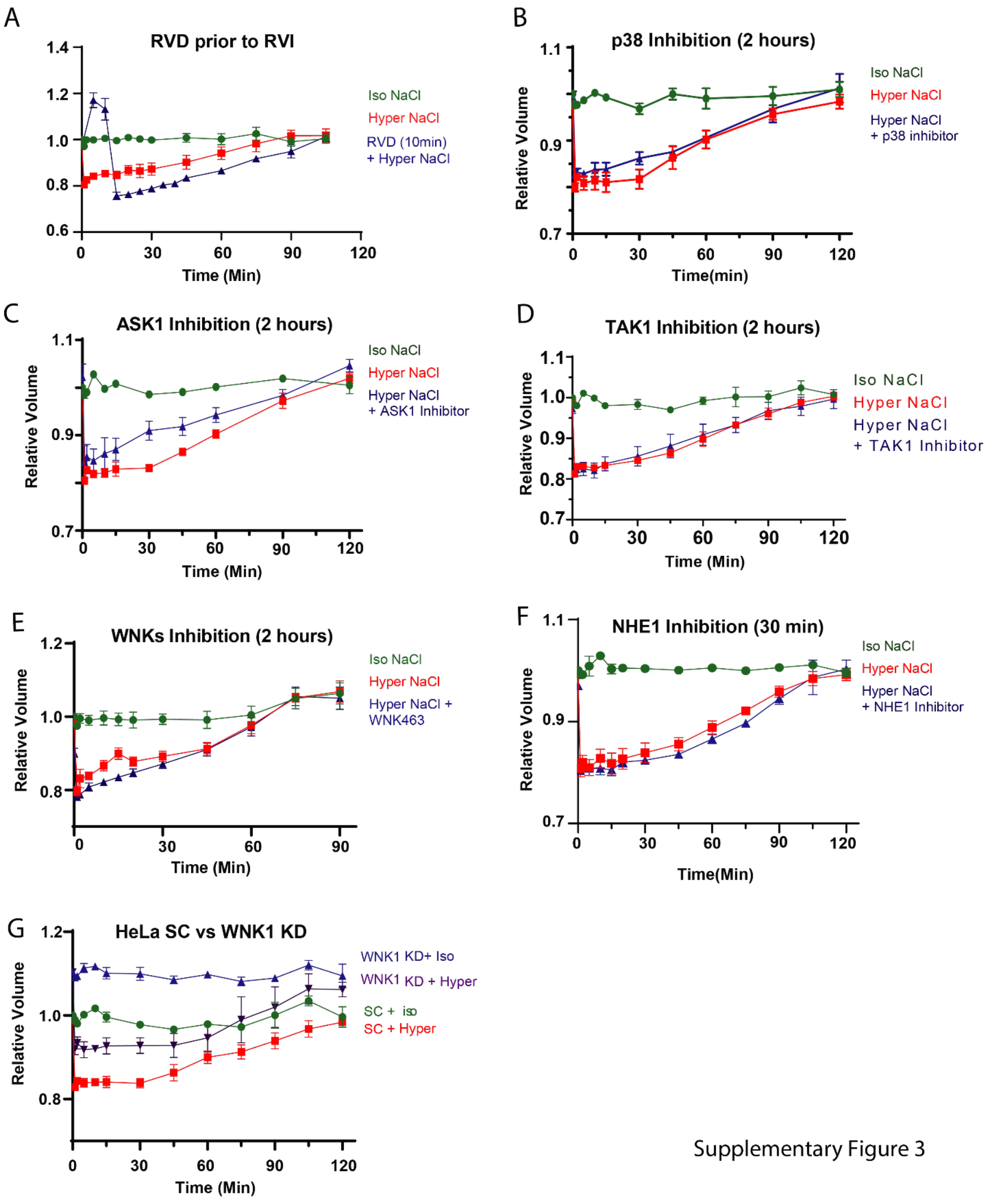

Supplementary Figure 3. Regulatory Volume Increase (RVI) in HeLa cells exposed to hypo-osmotic solution before hyper-osmotic shock, or treated with short-term p38, ASK1, TAK1, WNKs or NHE1 inhibitors.

(A) HeLa Scramble control (SC) cells were treated (or not) with hypo-osmotic NaCl solution for 10 minutes prior to switching to iso- or hyper-osmotic NaCl solution to analyze RVI. (**B-E**): 2 hours inhibition prior to measuring RVI under iso- or hyper-osmotic NaCl solution in the absence/presence of: **(B)** 5 μM SB203580 (p38 inhibitor), **(C)** 10 μM GS-444217 (ASK1 inhibitor), **(D)** 1 μM 5Z-7-Oxozeaenol (TAK1 inhibitor), **(E)** 10 μM WNK463 (pan-WNKs inhibitor). **(F)** 10 μM of Cariporid (NHE1 inhibitor) applied for 30 minutes. **(G)** HeLa scramble control (SC) or WNK1 KD cells were washed with PBS and exposed to iso- or hyper-osmotic solutions for the indicated times. Cell volume recovery (RVI) was measured as above. All data are mean±s.e.m, N≥3 independent experiments.

ORIGINAL BLOTS

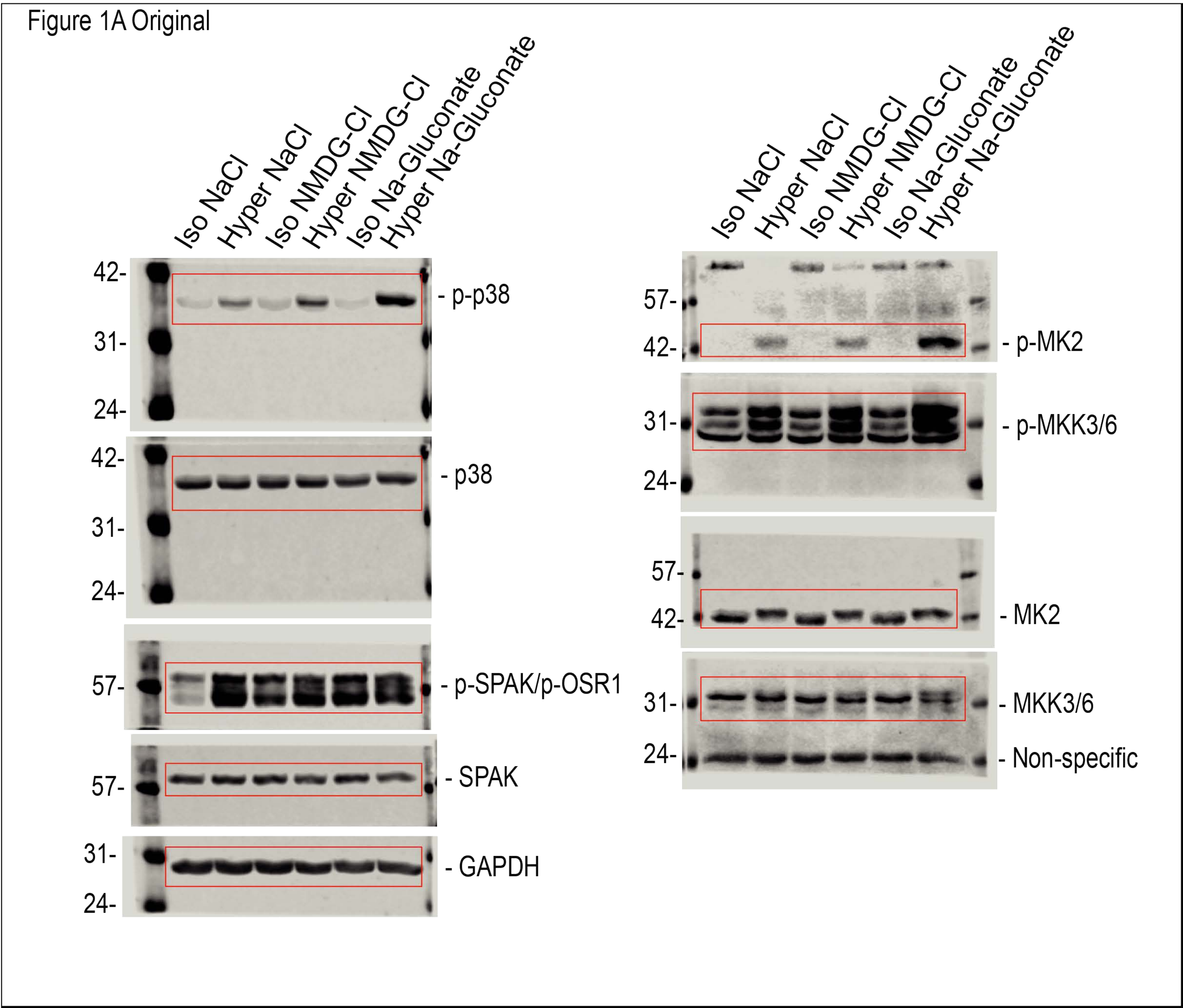

Figure 1B Original

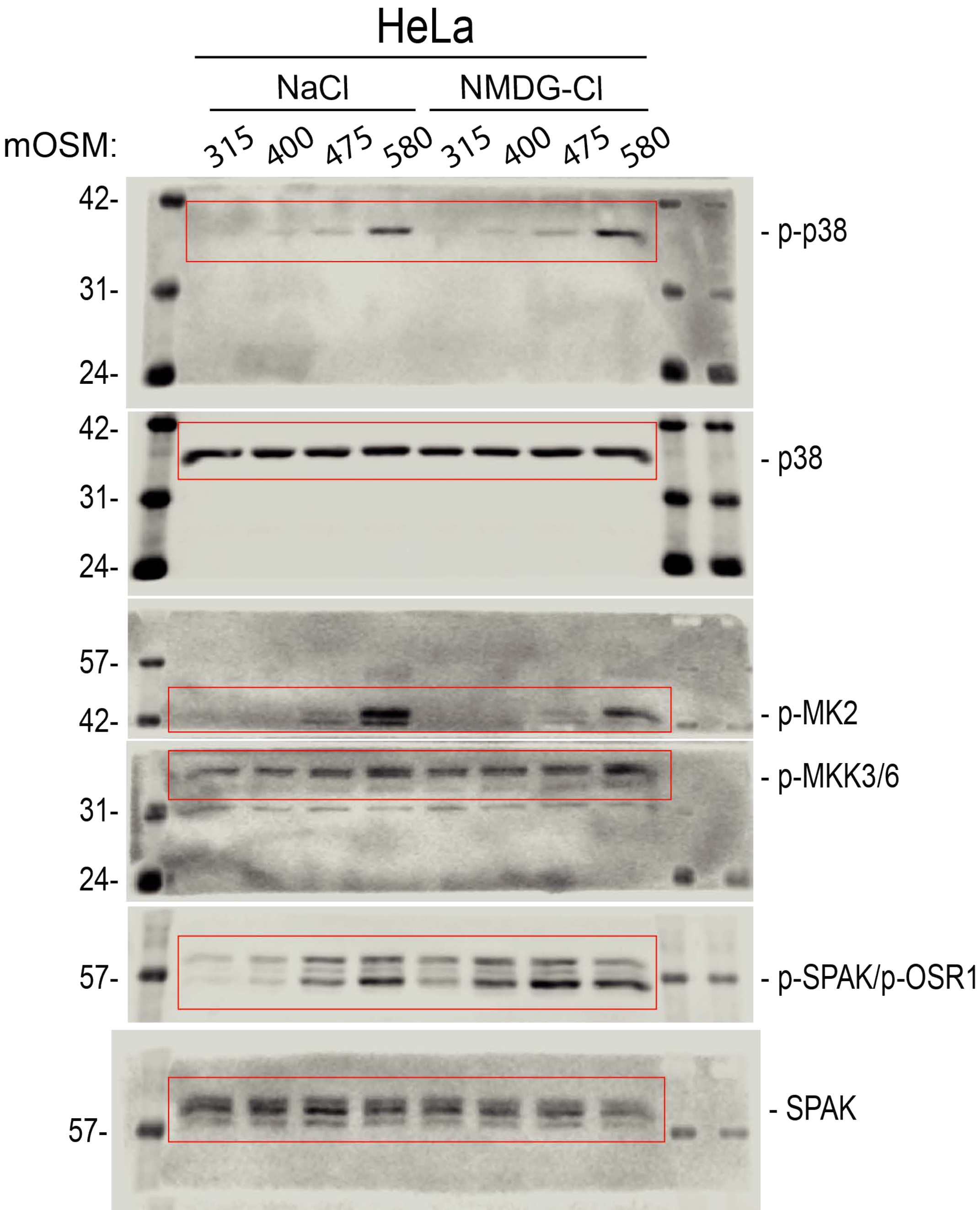

Figure 1D Original - 01

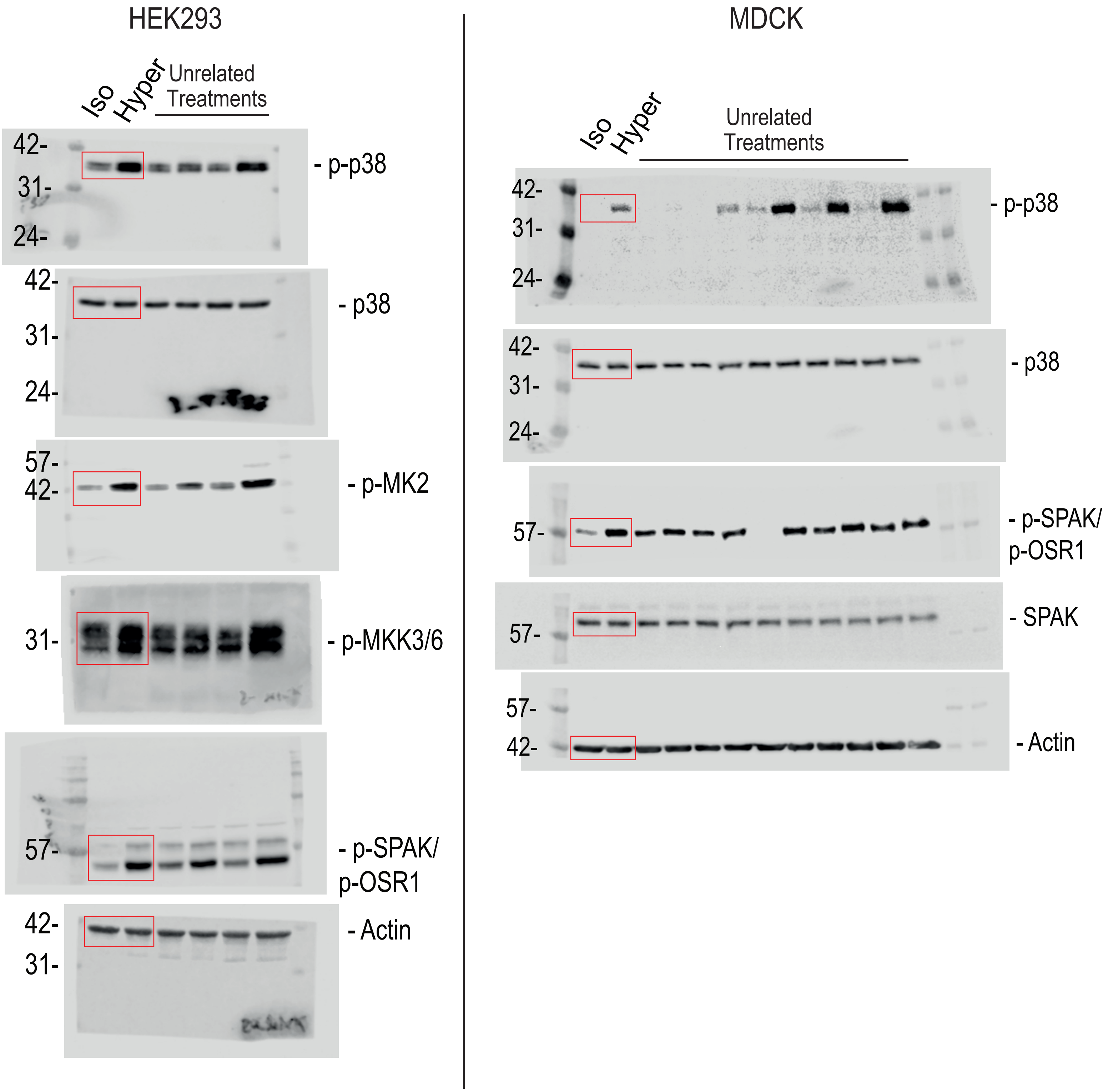

Figure 1D Original - 02

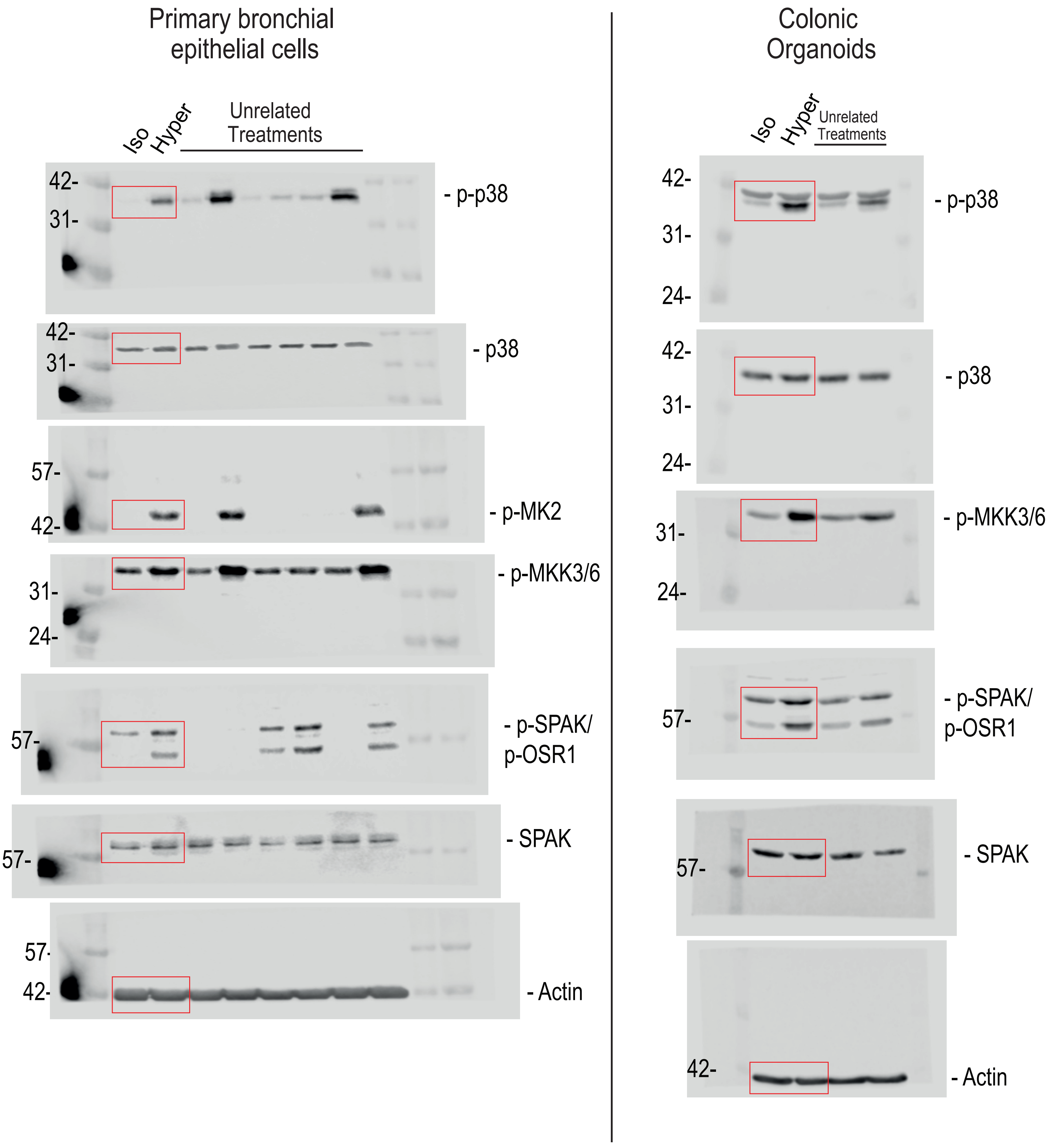

Figure 2A Original

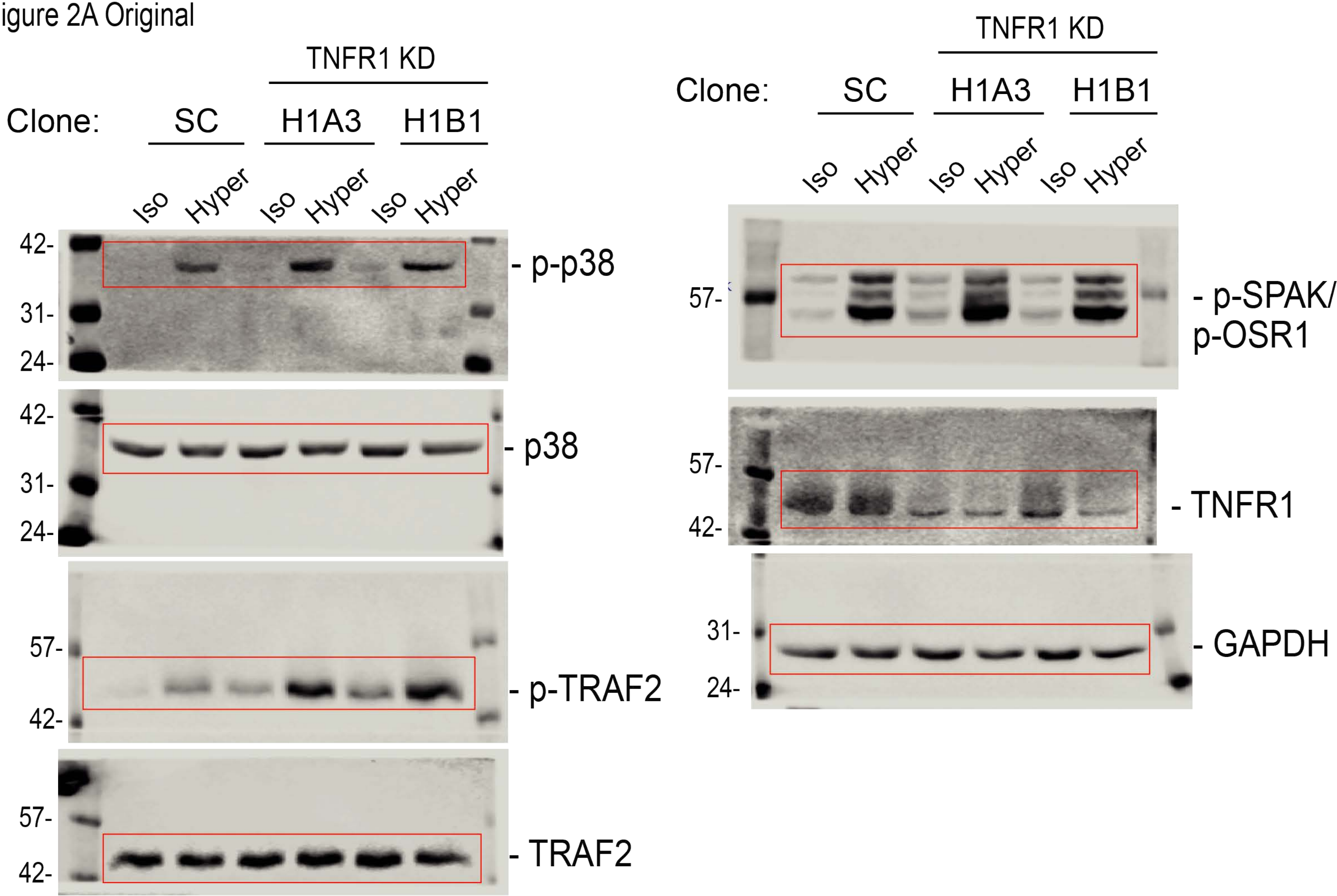

Figure 2B Original

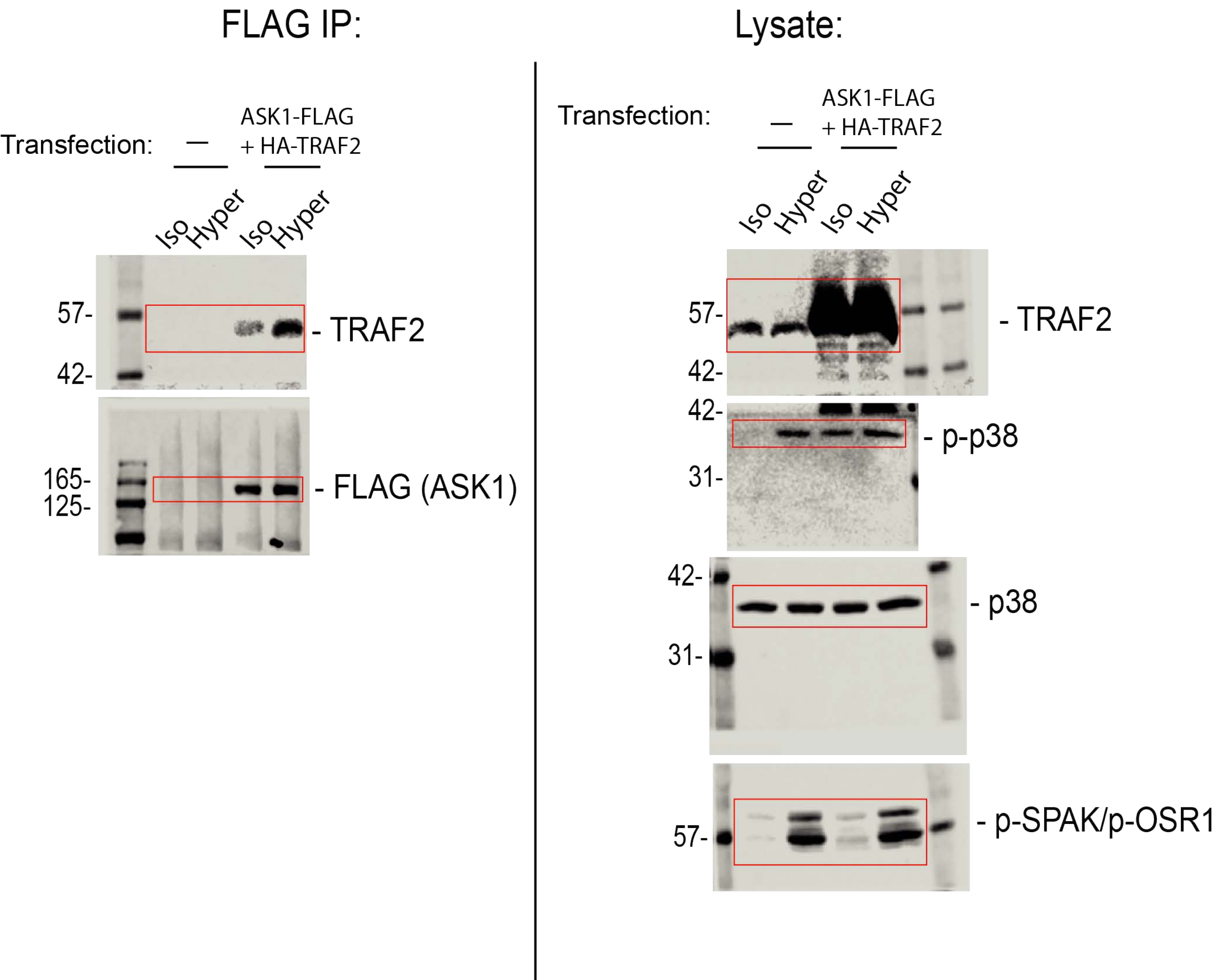

Figure 2C Original

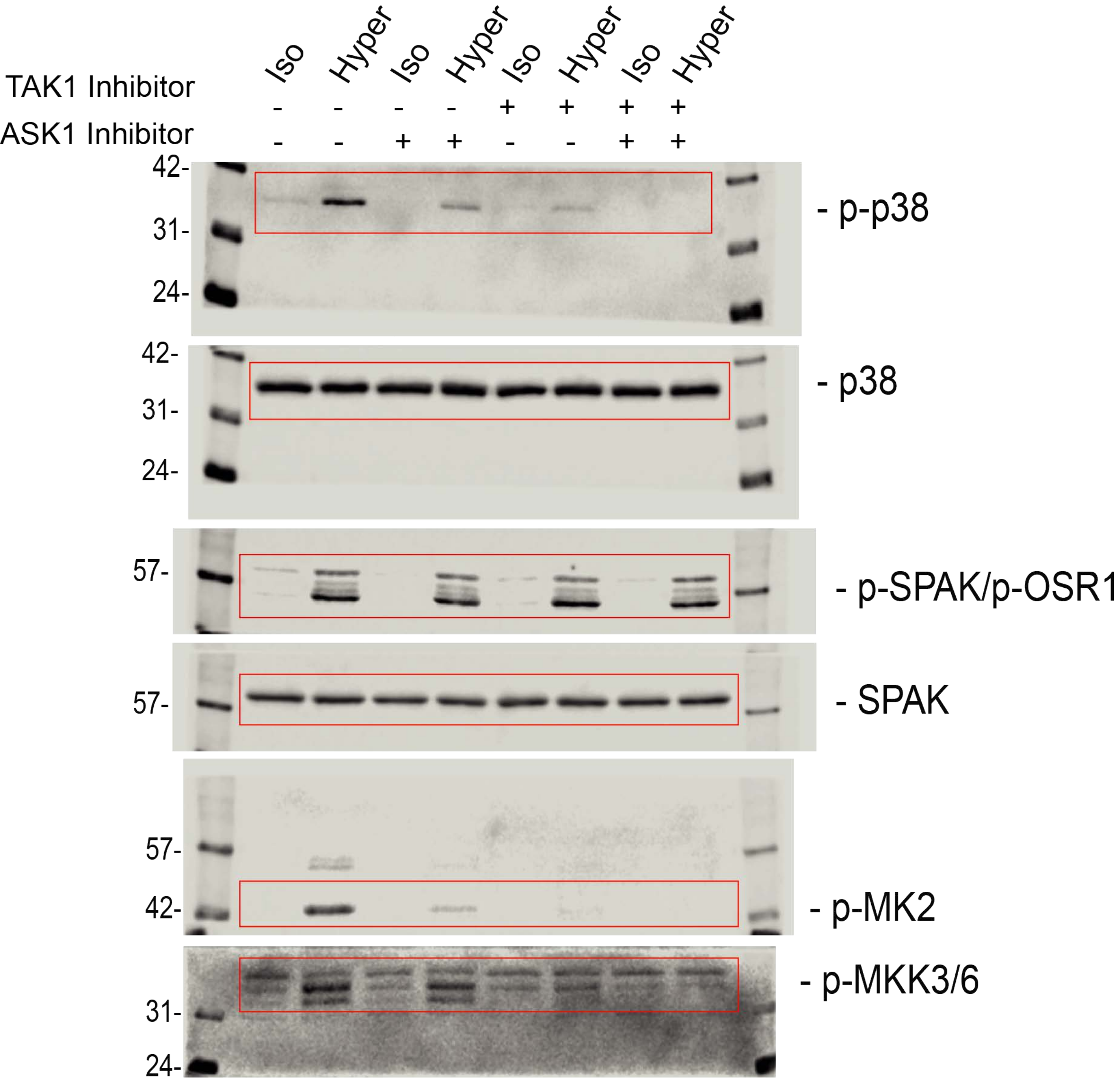

Figure 2D Original

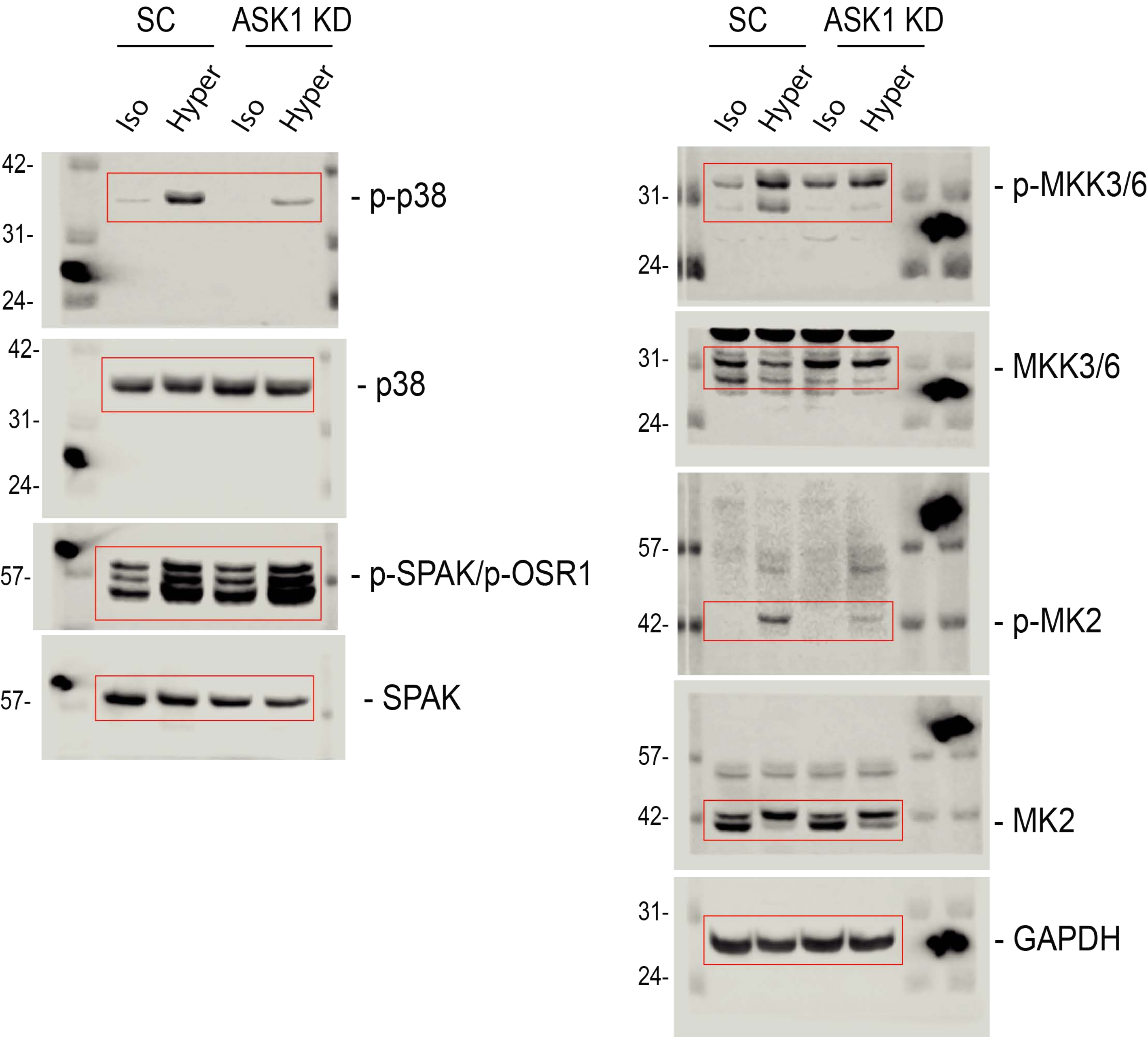

Figure 3A Original

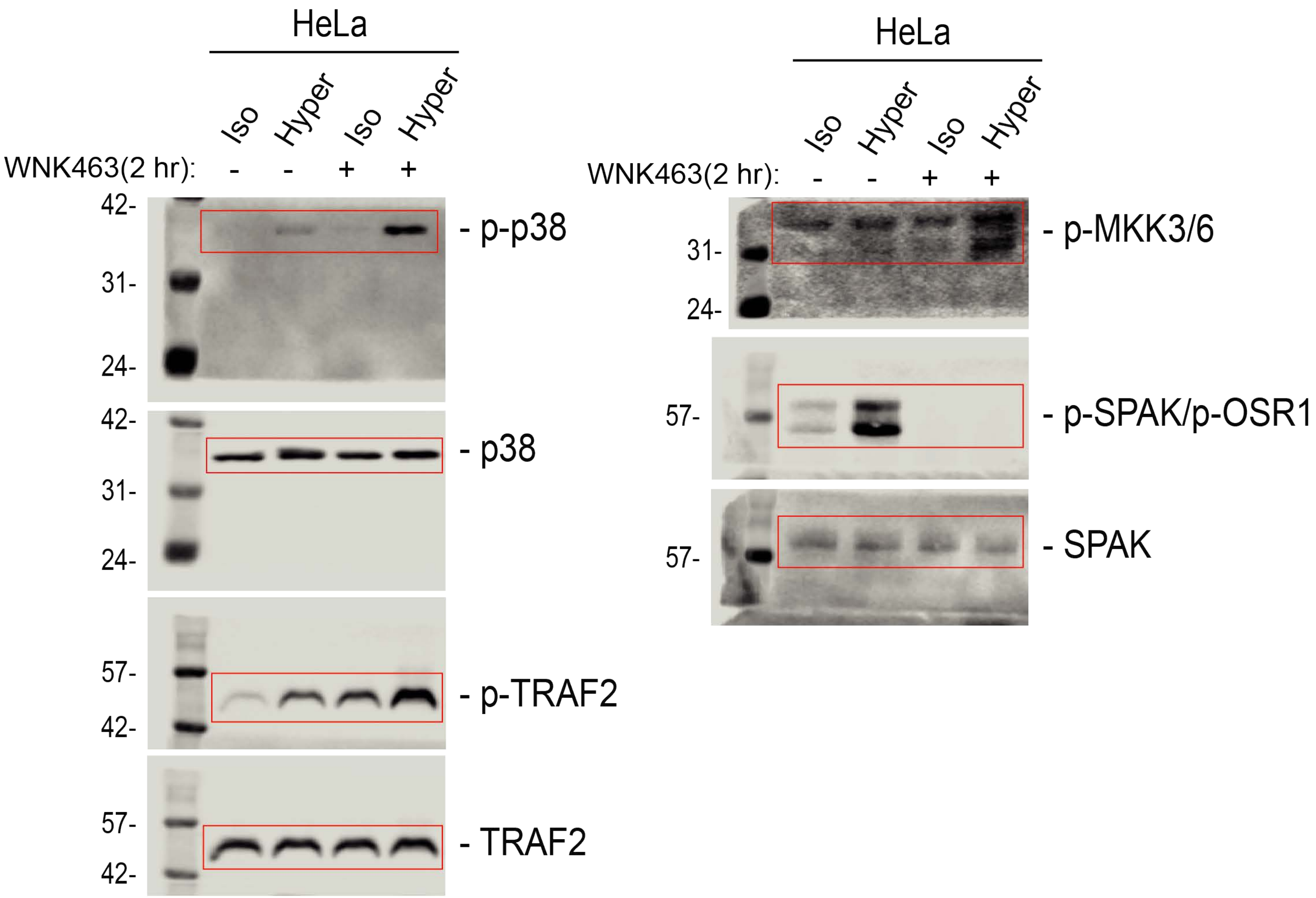

Figure 3B Original

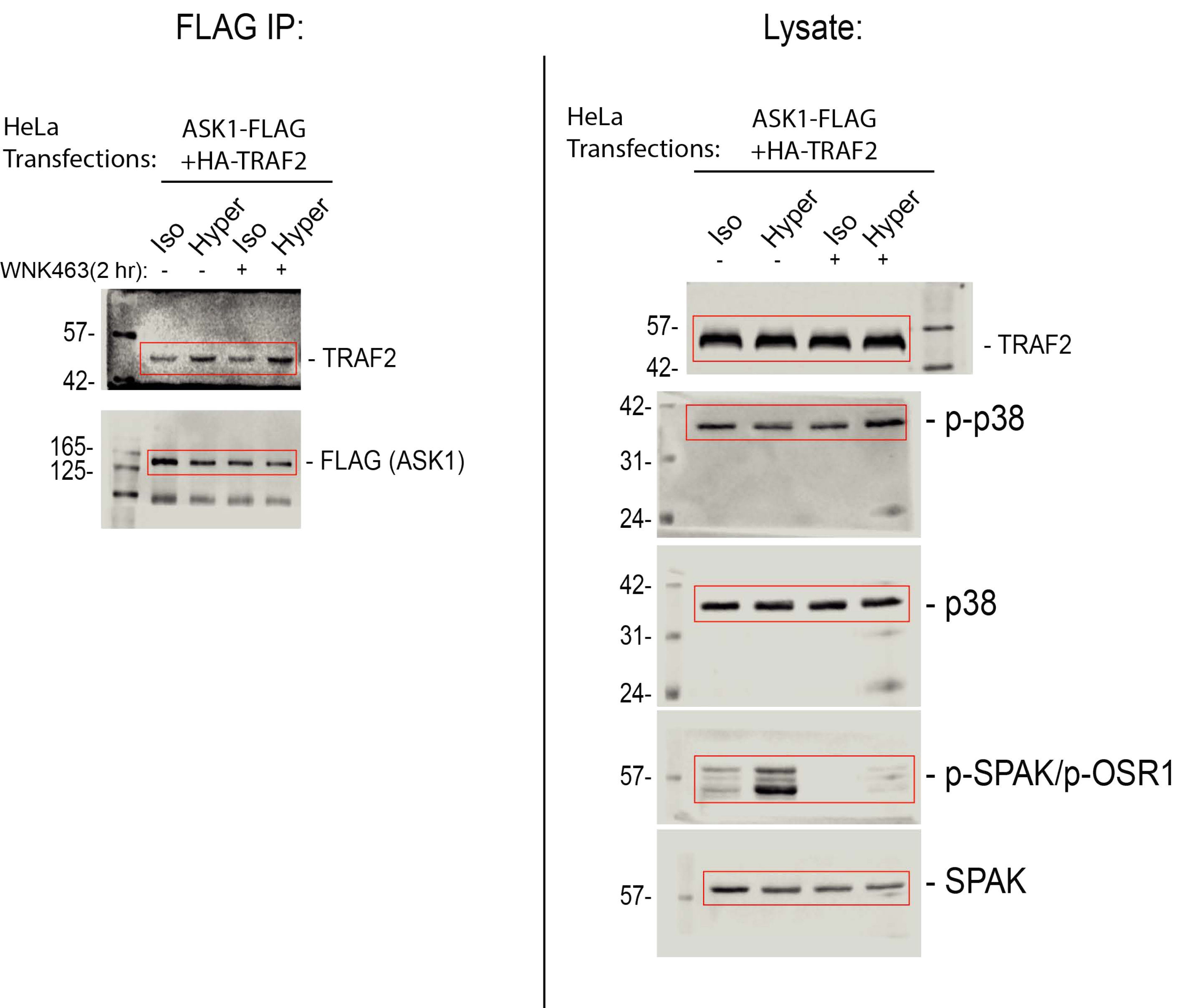

Figure 3C Original

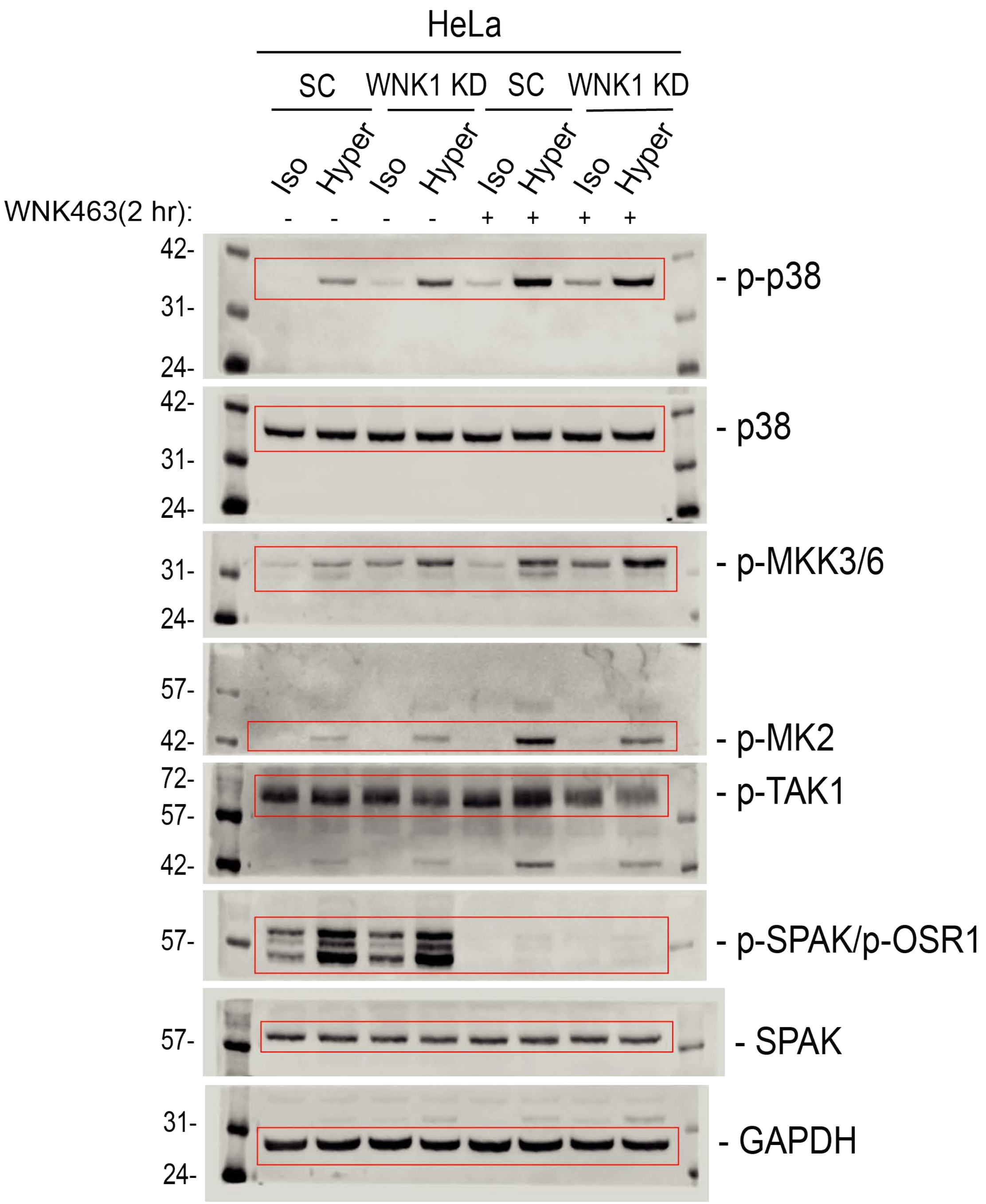

Figure 4A Original

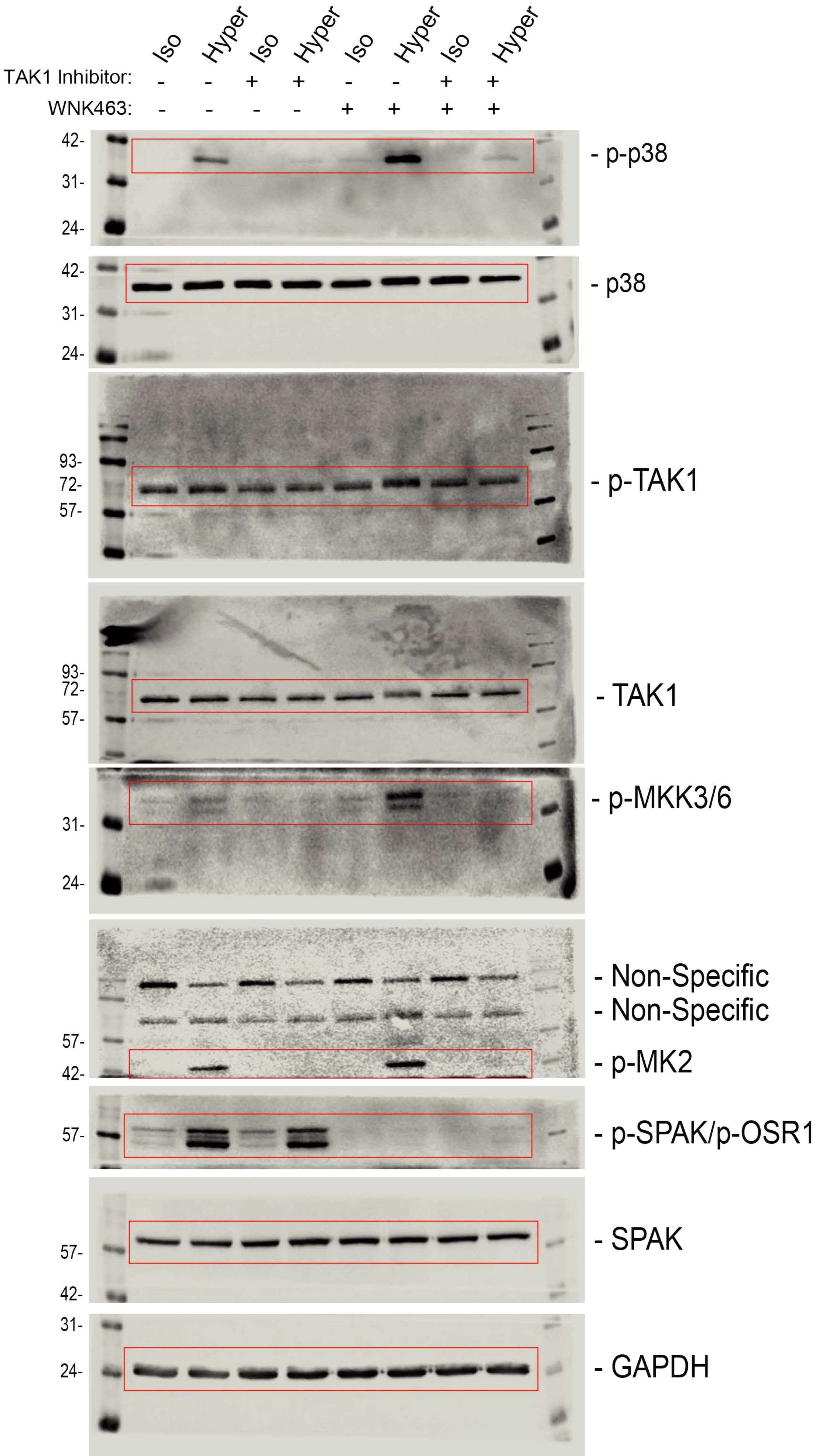

Figure 4B Original

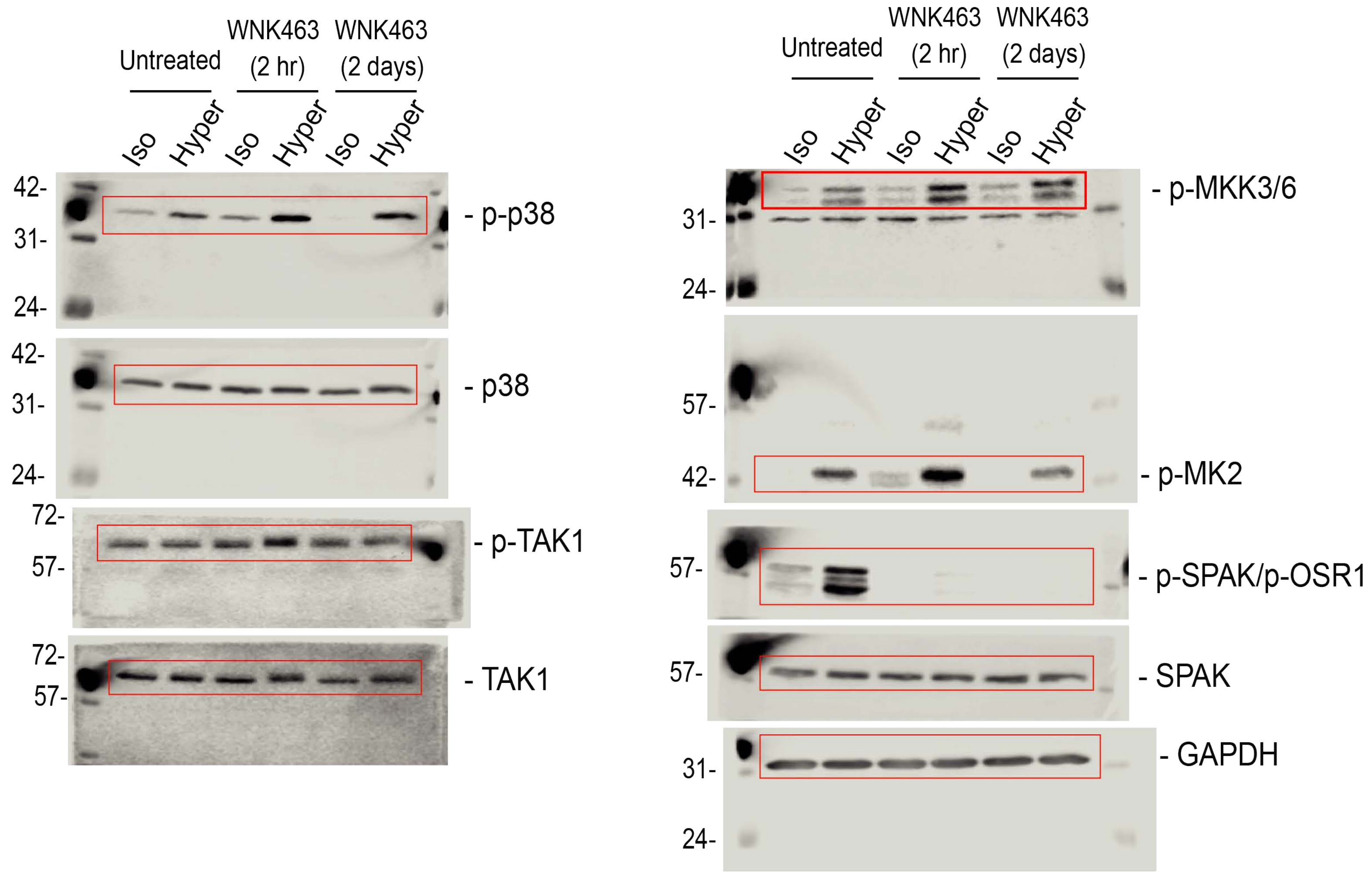

Figure 5C Original

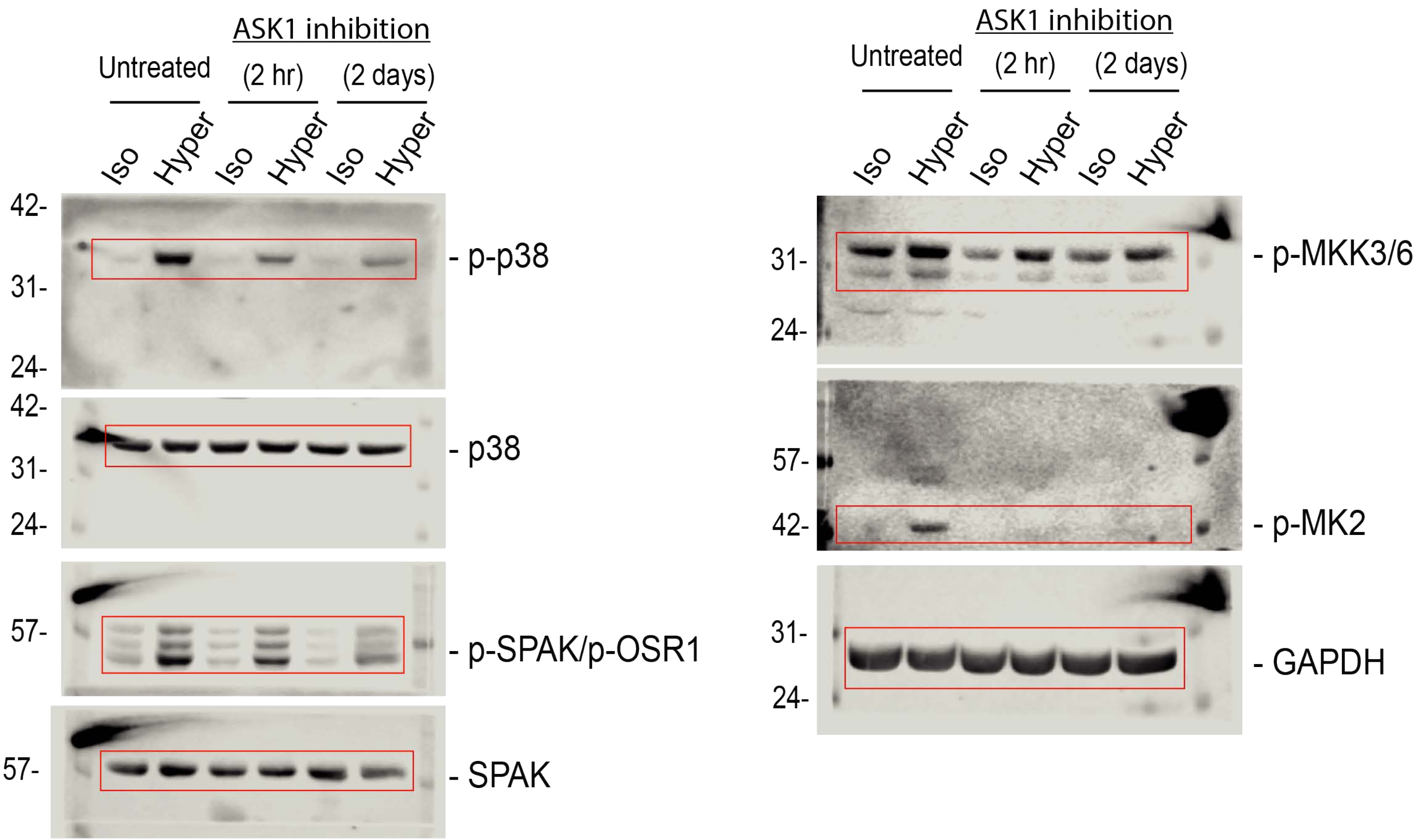

Figure 5E Original

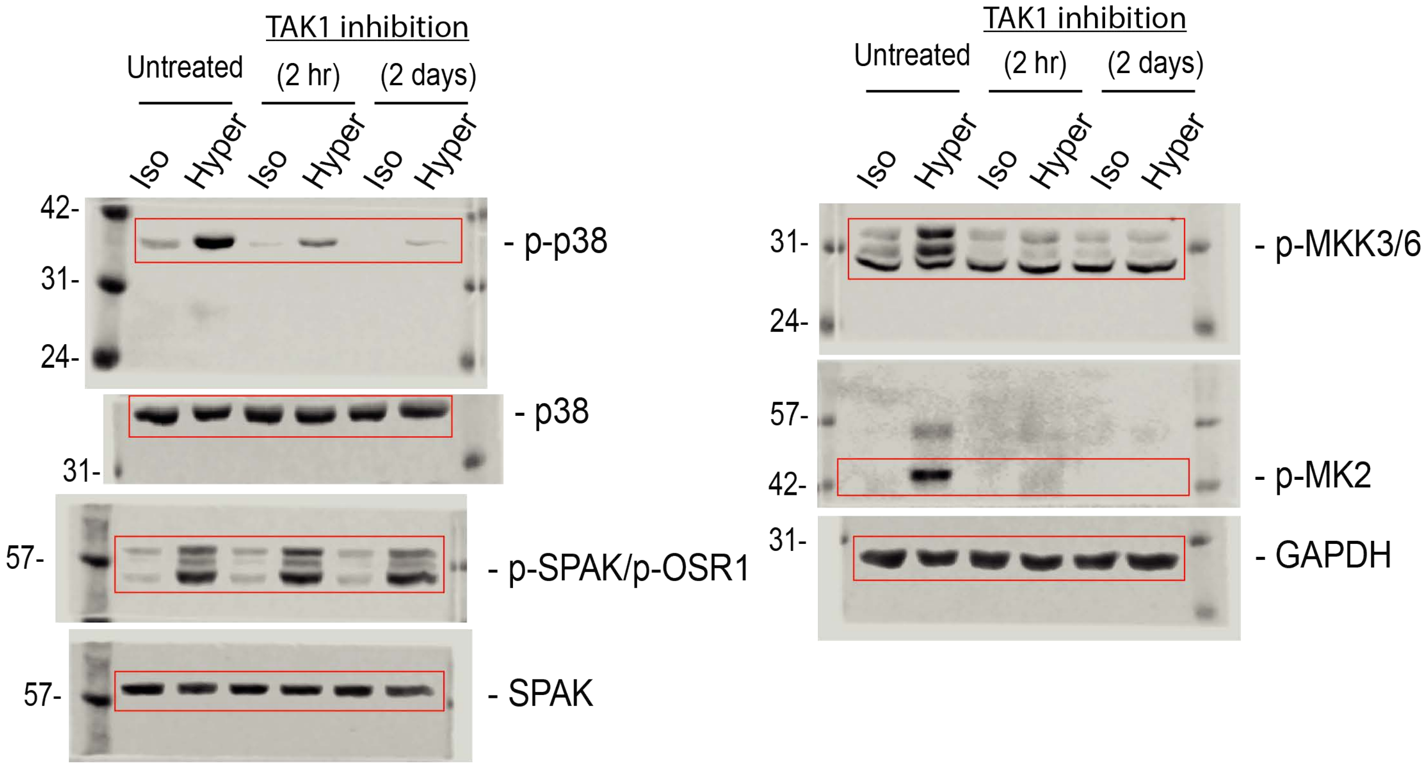

Figure 6A Original

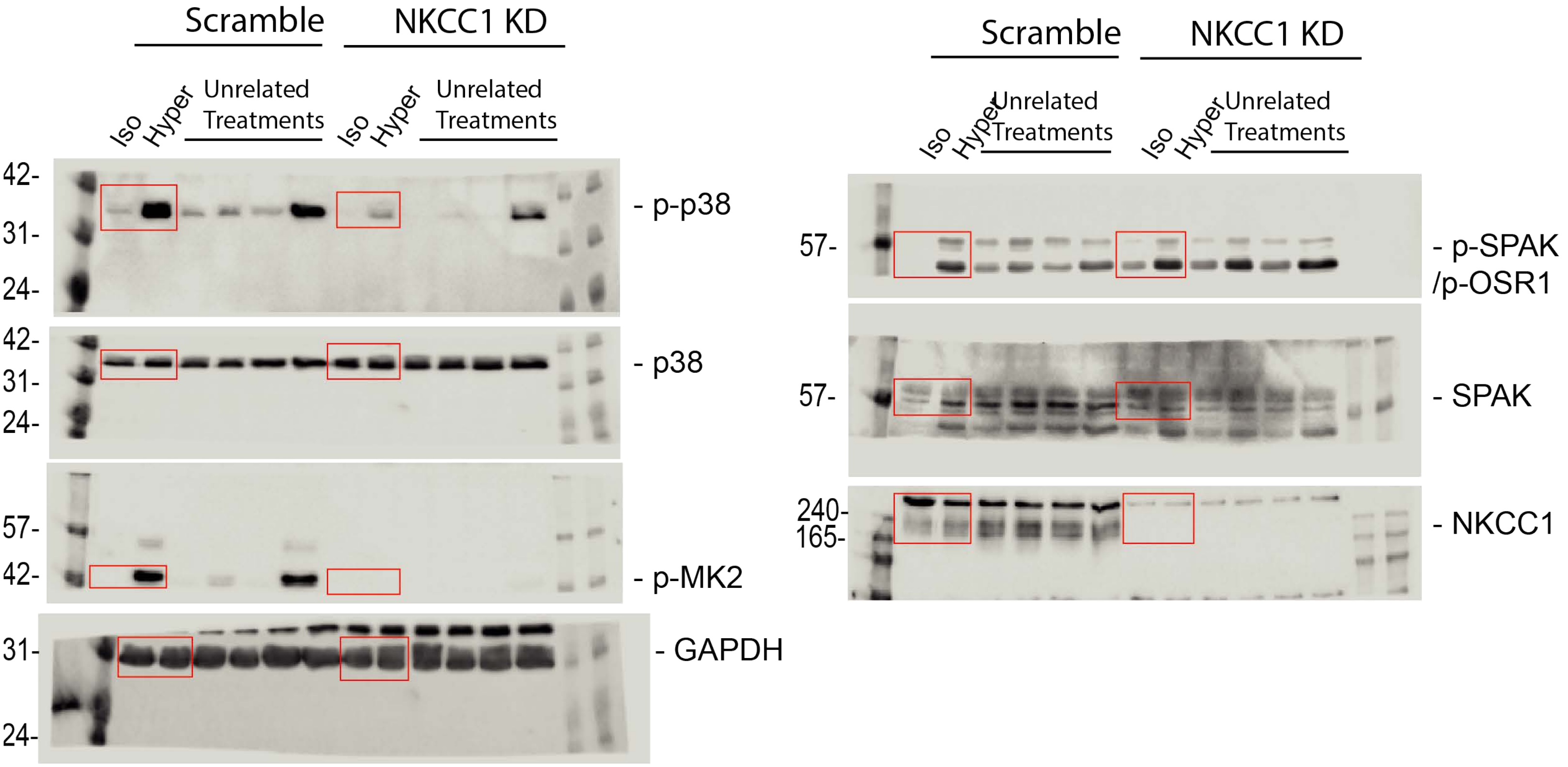

Figure 7C Original

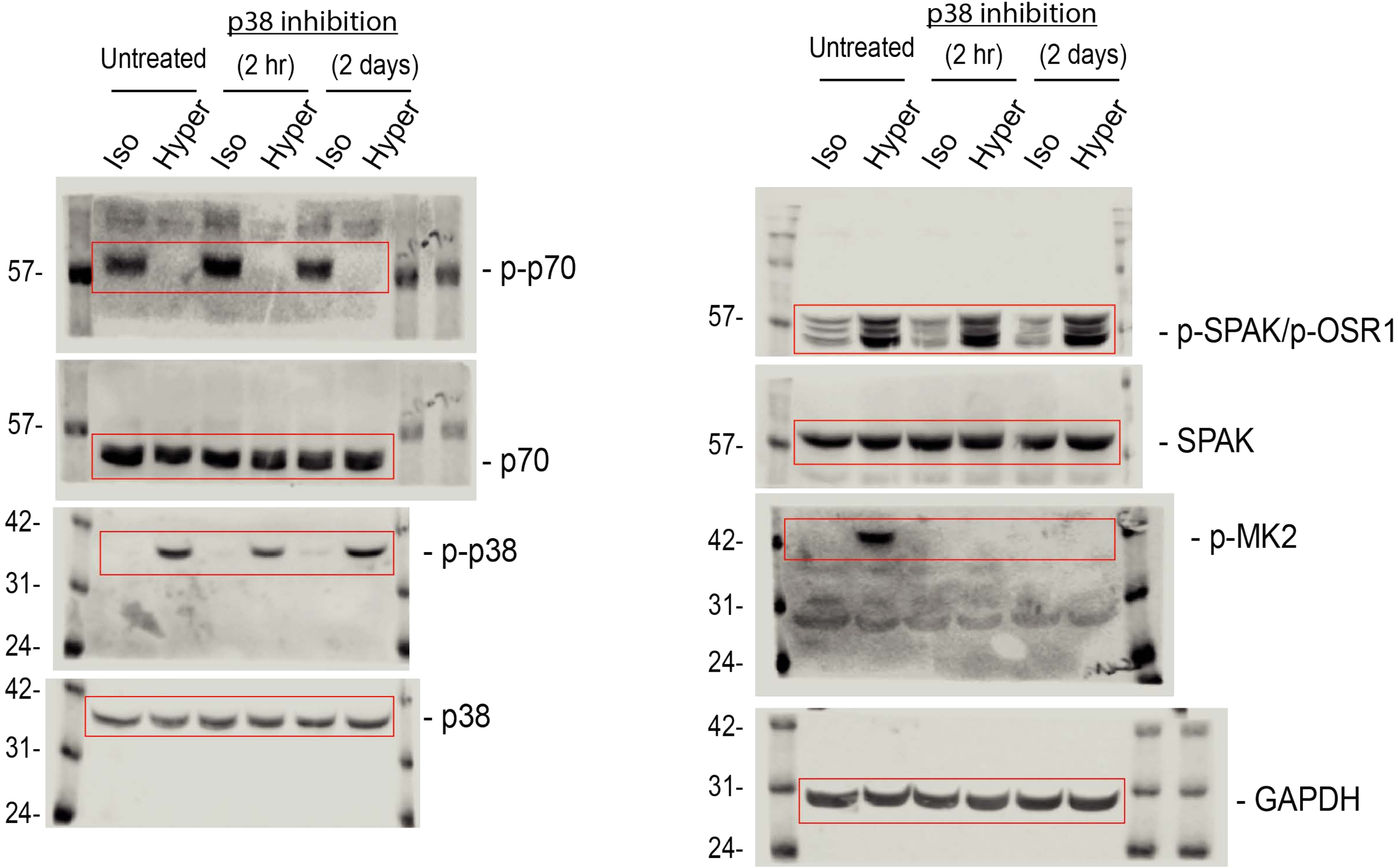

Figure S1A Original

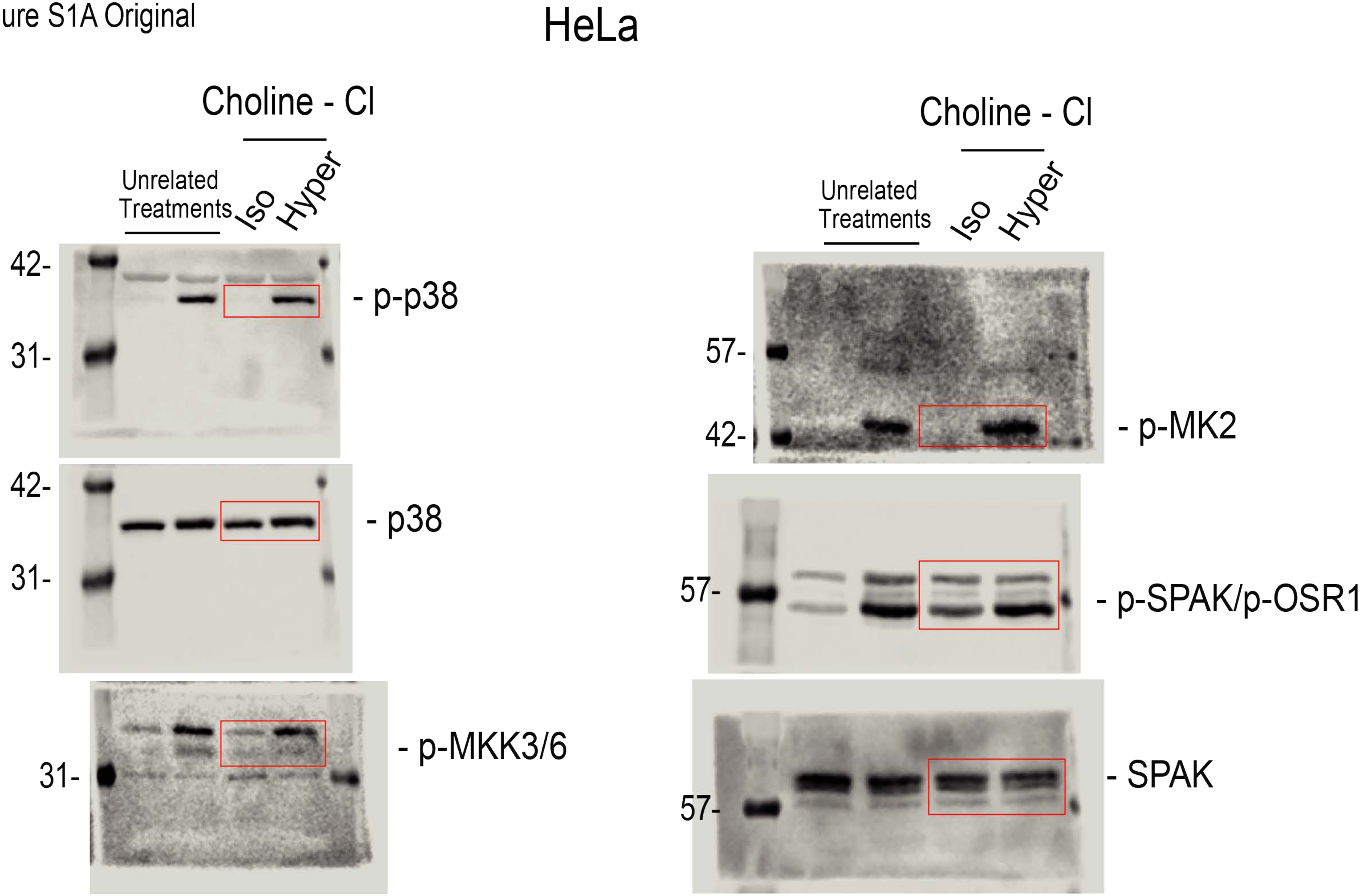

Figure S1B Original

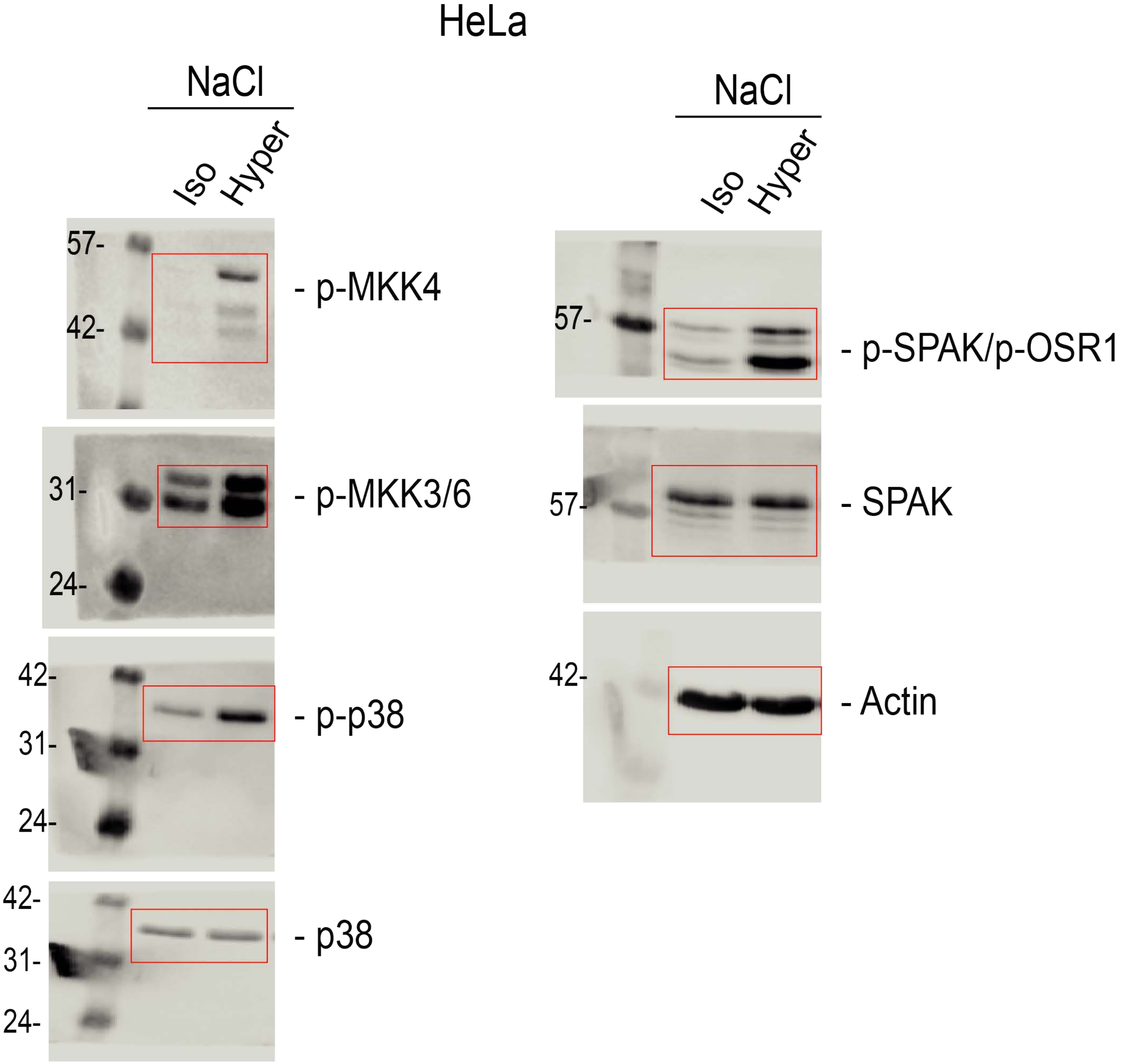

Figure S1D Original

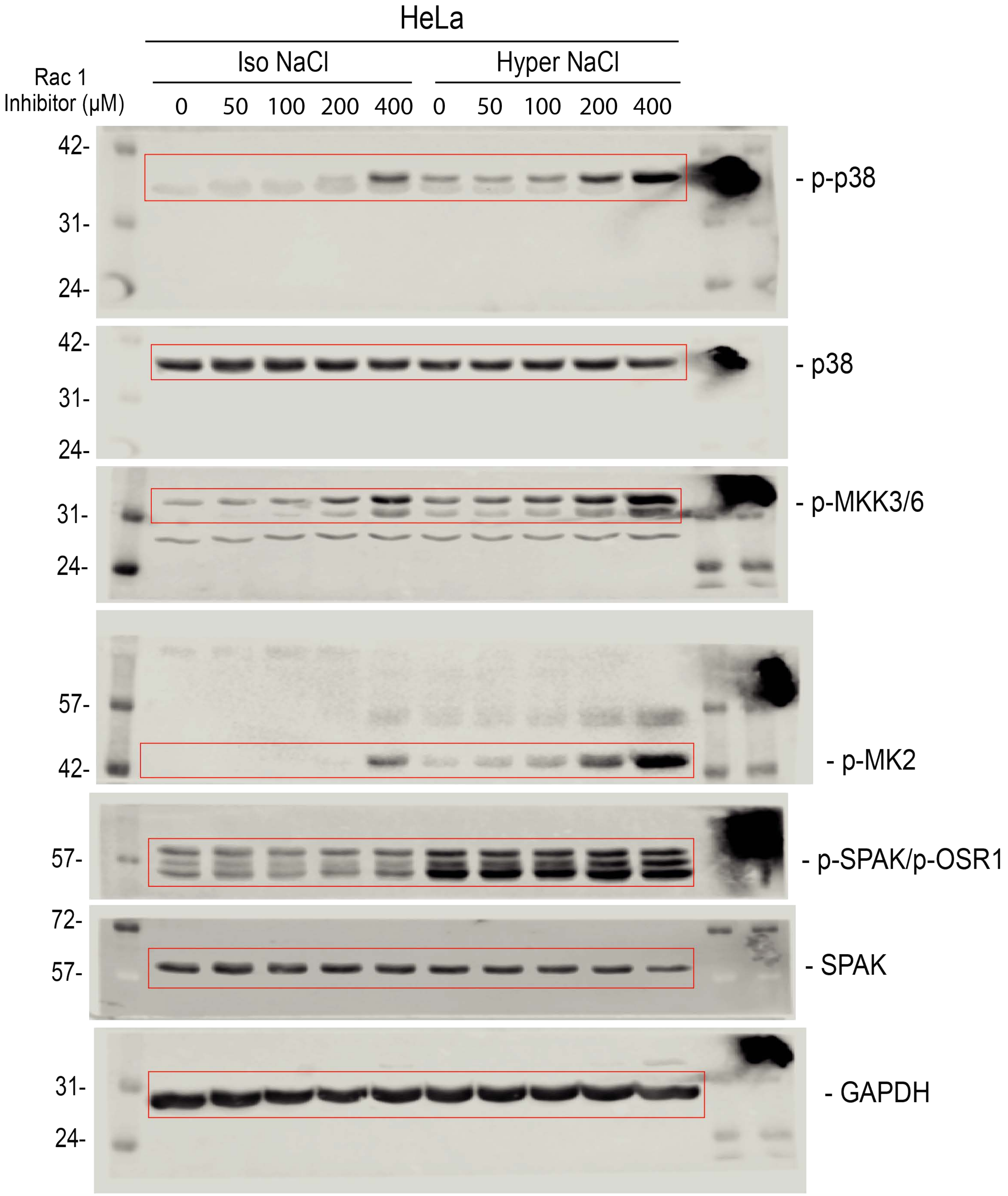

Figure S1E Original

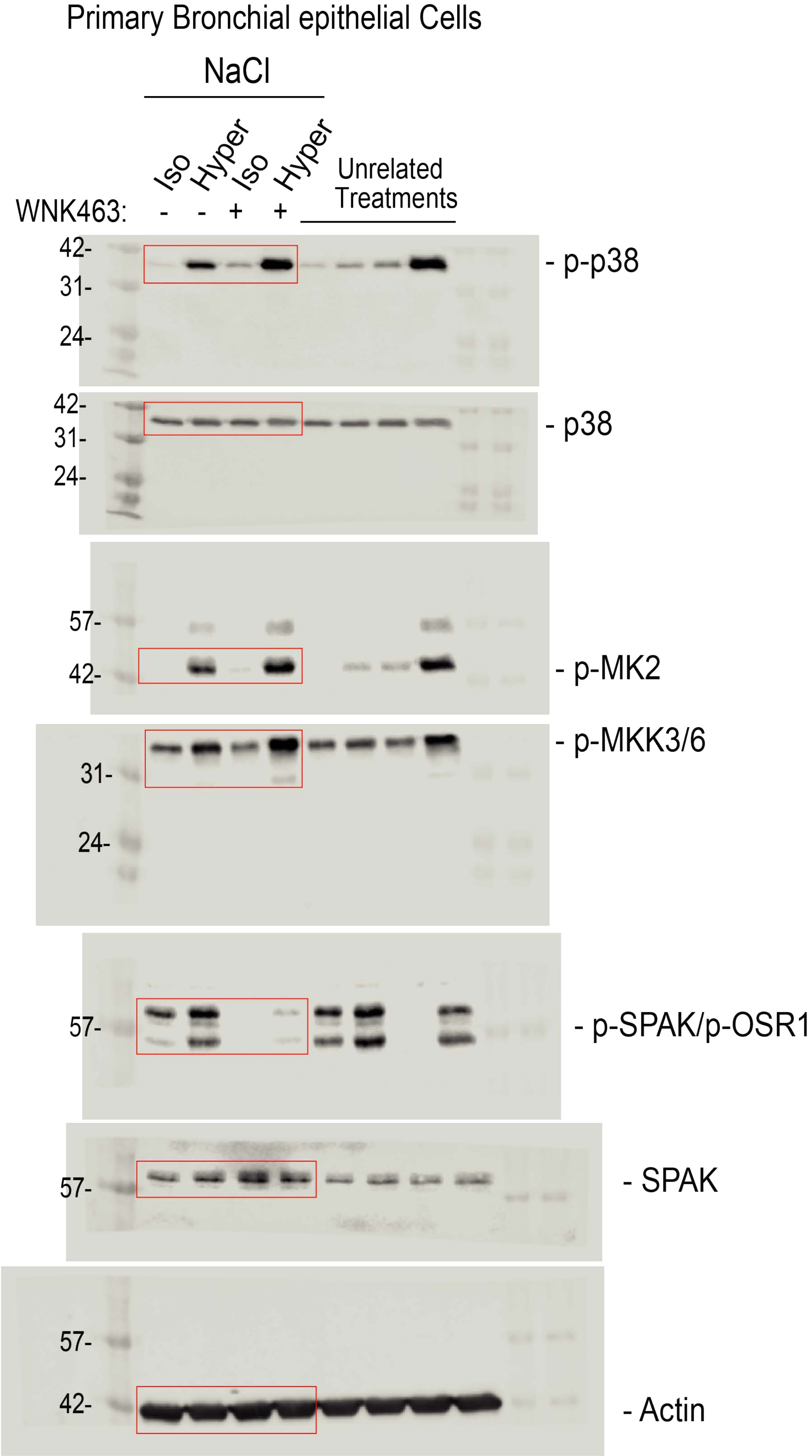

Figure S1F Original

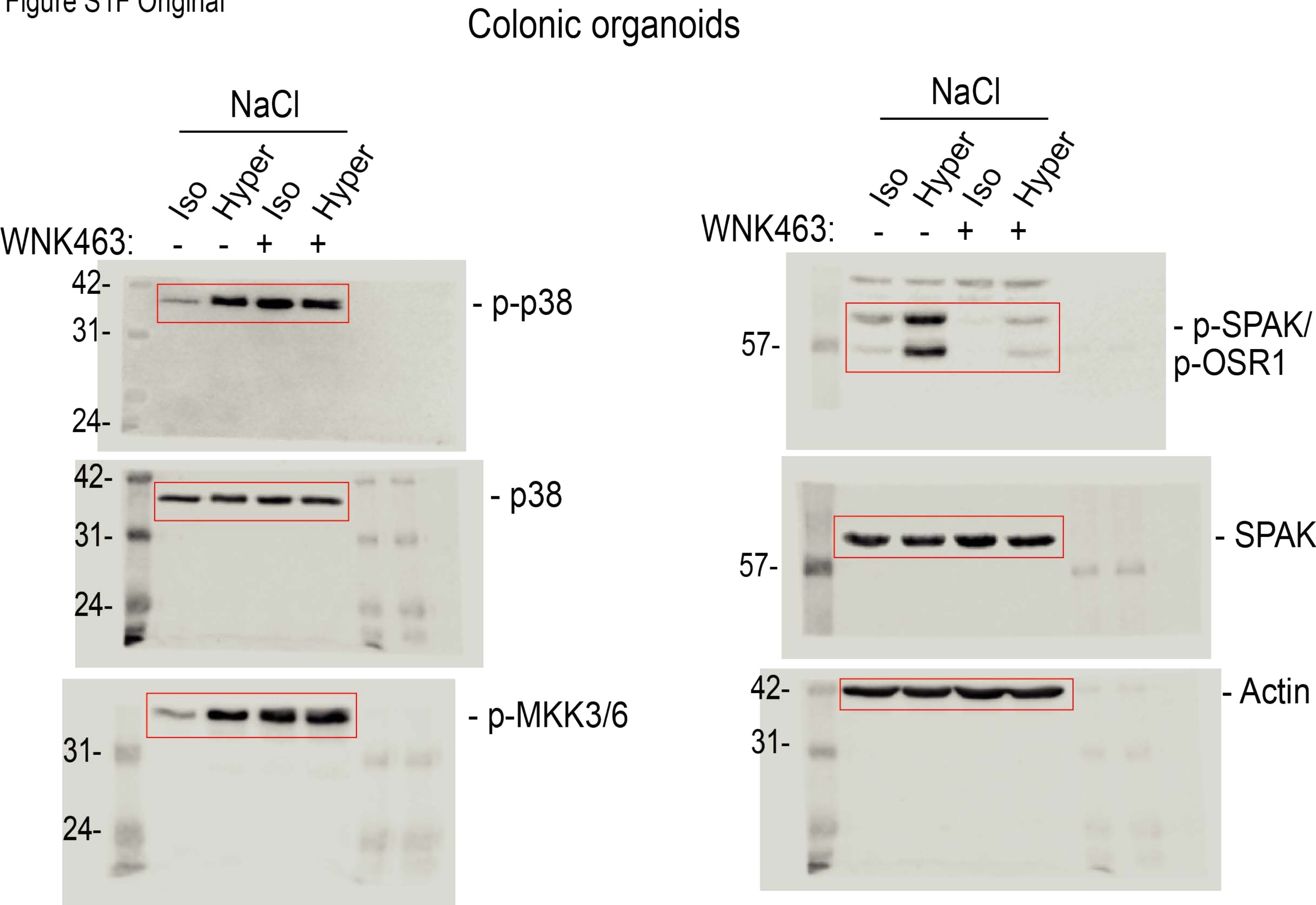

Supplement: Supplementary file 1 — Supplementary Information. [file 41598_2022_18630_MOESM1_ESM.pdf]
